# Impact of Heterovalent Cu^2+^ and Ag^+^ Doping on the Structural, Optoelectronic, and Photocatalytic Properties of ZnO for Enhanced Solar-Driven Hydrogen Evolution and Organic Pollutant Degradation

**DOI:** 10.1021/acsomega.5c05419

**Published:** 2025-09-15

**Authors:** Saedah R. Al-Mhyawi, Ahlam I. Al-Sulami, Fatimah Mohammad H. AlSulami, Reema H. Aldahiri, Merfat M. Alsabban, Fuad Mohammed A. B. Mosa, Jawza Sh Alnawmasi, Omer Nur, A. Rajeh, Mohammed A. Mannaa

**Affiliations:** † Department of Chemistry, College of Science, 441424 University of Jeddah, Jeddah 23218, Saudi Arabia; ‡ Ministry of Energy, General Director for International Project Management Office and Advisor of Energy Minister, Local Content and Risk Management, Jeddah 23218, , Saudi Arabia; § Department of Chemistry, College of Science, 89660 Qassim University, Buraydah, Qassim 51452, Saudi Arabia; ∥ Department of Science and Technology, 101031 Linkoping University, Campus Norrkoping, Norrkoping SE-60174, Sweden; ⊥ Physics Department, Faculty of Applied Science, Amran University, Sanaa 12344, Yemen; # Chemistry Department, Faculty of Science, Amran University, Sanaa 12344, , Yemen

## Abstract

The preparation of photocatalysts capable of harnessing solar light energy as a sustainable and renewable source for treating wastewater containing organic pollutants and for H_2_ production from water splitting represents a significant direction in the photocatalyst field. In this study, S-scheme CuO/Ag_2_O–ZnO heterostructures with different CuO contents were prepared by the sol–gel method. XRD results confirmed the doping of ZnO by Ag^+^ and Cu^2+^. Also, the intensity of peaks decreased obviously after the addition of Ag^+^, while the addition of Cu^2+^ was accompanied by an increase in the peak intensity. UV–visible analysis results displayed a significant red shift of the absorption edge to the visible region, and a remarkable reduction in the band gap of ZnO was detected after the addition of Ag^+^ and Cu^2+^. The photocatalytic performance was evaluated by photodegradation of MB, TC, and H_2_ evolution under sunlight illumination. The results revealed that the addition of Ag^+^ and Cu^2+^ enhanced the photocatalytic activity of ZnO significantly due to the formation of the S-scheme (*p*–*n*)-heterojunction heterostructure, which efficiently improved the separation and increased the lifetime of the photogenerated charge carriers compared with pure ZnO nanoparticles. The ESR and radical quenching results revealed that ^•^O_2_^–^ and ^•^OH are the active radicals in the photodegradation of methylene blue (MB) and tetracycline (TC). The photodegradation mechanism, mineralization (TOC), and kinetic degradation were studied. The CuO/Ag_2_O–ZnO heterostructure exhibited excellent photocatalytic activity, reusability, and stability.

## Graphical abstract

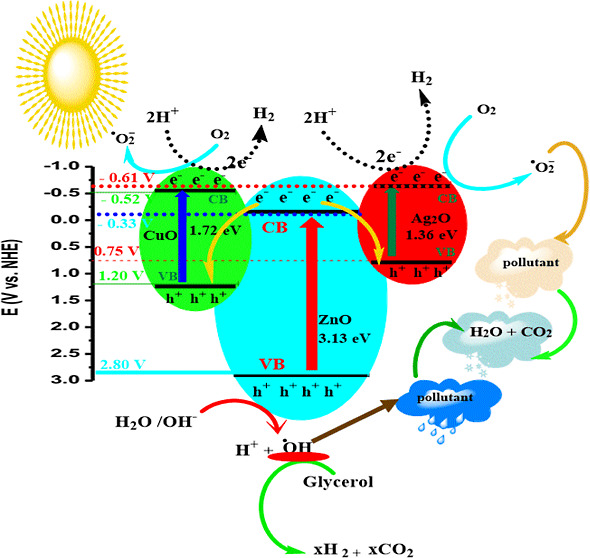

## 1 Introduction

In recent years, the global demand for energy and clean water has surged dramatically due to rapid population growth and economic development.^1,2^ The increase in energy demand has placed immense pressure on the present energy sources. Furthermore, severe environmental issues have appeared due to the pollution that results from the combustion of conventional energy sources, particularly fossil fuels including greenhouse gas emissions, air pollution, and climate change, which threaten ecosystems and human health worldwide.^3–5^ On the other hand, water pollution is one of the greatest challenges across the world due to increasing pollutant sources as chemicals, dyes, inks, and cosmetics factories, which are present in wastewater with different hazardous and dangerous contaminants, posing a threat to the health of humans and other organisms.^4,6–13^ In response to these challenges, scientists and researchers have exerted extensive efforts to solve these environmental problems and to meet the growing energy demand by exploring renewable, efficient, clean, and sustainable energy sources.^14–17^ Hydrogen is a green energy fuel and has recently been considered as a fuel of the future that can address the world’s growing energy demands and reduce the environmental problems that result from fossil fuels.^18–23^ Various techniques have been applied for hydrogen generation, and the photocatalysis method is considered as one of the most effective methods for hydrogen production from water splitting and for wastewater treatment.^1,20,24,25^ Different semiconductors are employed as photocatalysts for hydrogen production and for water pollutant removal.^13,21,26–28^ However, the main challenges that hinder the effective application of photocatalysts are their stability, low catalytic efficiency, low solar light responsiveness, and the high recombination rate of the photogenerated charges, which result from their wide band gap (*E*_g_).^29–31^ On the other hand, the fabrication of photocatalysts with an S-scheme heterojunction-heterostructure has garnered significant attention in recent years, primarily attributed to their distinctive properties as highly effective inhibitors of e^–^/h^+^ recombination, coupled with their superior oxidation (OP) and reduction (RP) potentials, which contributed to enhancement of their photocatalytic efficiency.^32,33^ Moreover, the fabrication of S-scheme *p*–*n* heterojunction photocatalysts is considered to be the most effective compared with other types of heterostructures.^32^ In comparison to other semiconductors, ZnO is an n-type semiconductor and has attracted considerable attention in a wide range of applications due to its advantageous properties such as high photoreactivity, physical and chemical stability, controlled morphologies, low cost, and high redox potential.^34,35^ Also, ZnO is considered a promising material in optoelectronic materials and as an industrial catalytic material. However, ZnO has some disadvantageous that restrict its photocatalytic reactivity such as a wide band gap (∼3.2 eV), rapid e^–^/h^+^ pair recombination, and low visible light response. Different techniques have been applied for improving the photocatalytic activity of ZnO and enhancing its utilization of sunlight energy (especially the visible region), including doping, decoration, and combining metal, nonmetal ions, and other semiconductors.^4^ On the other hand, the doping of ZnO by transition metals leads to formation of a heterojunction structure with a narrow band gap due to the creation of a new energy state in the energy gap of ZnO, which in turn enhances the response of ZnO in the visible region.^34^ Additionally, coupling ZnO with narrow band gap semiconductors enhanced the high utilization of visible light and effectively restrained the recombination of e^–^/h^+^ pairs. Copper oxide (CuO) and silver oxide (Ag_2_O) are important transition metal oxides and have a narrow band gap of about 2.0 and 1.2 eV for CuO and Ag_2_O, respectively, indicating the excellent capability of these two metal oxides to absorb visible light.^7,36^ CuO and Ag_2_O are both p-type materials and have garnered a lot of interest in various applications because of their distinct optical and electrical characteristics.^4,37,38^ Binary CuO/Ag_2_O is not sufficient to achieve optimal photocatalytic reactivity due to issues like rapid recombination of photogenerated electron–hole pairs and limited stability and reusability. However, to overcome these limitations, ZnO is the most appropriate n-type semiconductor that can be added to CuO/Ag_2_O to create a ternary CuO/Ag_2_O–ZnO heterostructure. ZnO offers several advantages that make it a valuable component in ternary CuO/Ag_2_O–ZnO photocatalysts. It significantly suppresses the recombination of photogenerated electron–hole pairs.^8,32^ Several properties have been determined after the addition of CuO into Ag_2_O–ZnO nanoparticles, including (i) formation of *p*–*n* heterojunctions between p-type CuO/Ag_2_O and n-type ZnO, which improve charge separation and reduce the recombination rate of photogenerated charges efficiently;^37^ (ii) creation of narrow band gaps which shifted the optical absorption of Ag_2_O–ZnO into the visible region;^39^ and (iii) improvement of the reusability and stability of the prepared photocatalyst. The most significant disadvantages are the environmental impacts, toxicity, and ions leaching into aqueous environments. This can reduce their long-term stability and photocatalytic performance and also poses environmental risks. However, several approaches have been applied to overcome or minimize these drawbacks, including determining the appropriate calcination temperature and preparation method.

In this study, we prepared CuO/Ag_2_O–ZnO heterostructure-heterojunction (*p*–*n*) photocatalysts with different amounts of CuO by the sol–gel method. The effects of Ag^+^ and Cu^2+^ on the structural, optoelectronic, and photocatalytic properties of ZnO were investigated. The photocatalytic activity of pure and doped ZnO was examined by photodegradation of MB and TC under neutral sunlight. Additionally, the photoactivity of the samples was investigated by H_2_ evolution. The obtained results demonstrated that the formation of a *p*–*n*-heterojunction heterostructure significantly enhanced the photocatalytic activity of ZnO by improving the separation and increasing the lifetime of the photogenerated charge carriers compared with pure ZnO nanoparticles.

## 2 Experimental Section

### 2.1 Synthesis of Samples

A series of CuO/Ag_2_O–ZnO heterostructures were prepared by using the sol–gel method. First, 2 g of CTAB was dissolved completely in 50 mL of ethanol (solution 1). After that, 8 g of zinc acetate (Zn­(CH_3_COO)_2_·2H_2_O) was dissolved in 50 mL of an aqueous solution (25 mL of DI water and 25 mL of ethanol) under magnetic stirring, heated at 60 °C until the solution became clear, and then added to solution 1. Next, 0.05 g of AgNO_3_ was dissolved in 20 mL of water and then transferred to the above solution. Then, the required concentration of Cu­(NO_3_)_2_ was dissolved in 20 mL of water and then transferred to solution 1. The obtained solution was stirred magnetically for 2 h. After that, 10 mL of a NaOH solution with 2 M was added under continuous stirring until the gel appeared. Afterward, the gel was left overnight and then filtered, washed with DI water, and dried at 80 °C overnight. Finally, the resulting precipitate was calcined at 500 °C for 2.5 h. Pure ZnO and Ag_2_O–ZnO nanoparticles were prepared by the same procedures without adding Cu­(NO_3_)_2_ in the case of the Ag_2_O–ZnO sample and Cu­(NO_3_)_2_ and AgNO_3_ in the case of ZnO nanoparticles.

### 2.2 Sample Characterization

The crystal structure phases of the prepared samples were investigated by powder X-ray diffractometer (XRD, a PW Philips 1830) using Cu Kα radiation (λ = 1.5418 Å). The morphology of the photocatalyst samples was characterized by TEM using a JEOL-JEM-2100. For identification of chemical species, the samples were measured on a Fourier transform infrared spectrometer JASCO (Nicolet iS10, USA) via the KBr particle method. The chemical state and compositions were investigated by X-ray photoelectron spectroscopy (XPS, Axis Ultra DLD, Kratos) with a monochromatic Al Kα source at 1486.7 eV. The diffuse reflectance spectra (DRS) of the samples were obtained by using a Shimadzu spectrophotometer (UV3600, Japan). Photoluminescence (PL) spectra were obtained using a JASCO F·P.e750 Model (Japan) spectrofluorometer at an excitation wavelength of 325 nm.

### 2.3 Photocatalytic Activity Measurements

#### 2.3.1 Photodegradation of Organic Pollutants

The photodegradation reactions of MB and TC were carried out under sunlight illumination on sunny days from 11.00 a.m. to 2.00 p.m. In each test set, 50 mg of the photocatalyst was dispersed in 50 mL of the pollutant solution (20 mg·L^–1^). The photochemical reactor was surrounded by a cooling-water system. The mixture was magnetically agitated for 30 min in the dark and then irradiated under sunlight with continuous stirring. At designated intervals of illumination, 2 mL of the sample was collected and centrifuged to separate the photocatalyst powder and then analyzed using a UV–vis spectrophotometer (Shimadzu, MPC-3100). The following equation was used to determine the percentage of photodegradation^40,41^

1 $$\%\;photodegradation=\left(\frac{C_{o}-C_{t}}{C_{o}}\right)\times 100$$

where *C*_0_ and *C*_t_ represent the pollutant’s initial and equilibrium concentrations (mg/L).

Additionally, various radical scavengers, such as Na_2_EDTA, isopropanol (IPA), and benzoquinone (BQ), were administered as scavengers of h^+^, ^•^OH, and ^•^O_2_^–^, respectively, at a concentration of 1 mM in order to identify the active species radicals and the photodegradation mechanism of MB and TC over the CuO/Ag_2_O–ZnO heterostructure.

#### 2.3.2 Photocatalytic H_2_ Evolution Activity

The photocatalytic efficacy of the fabricated photocatalysts was evaluated for the production of hydrogen from water splitting under solar simulator irradiation using a 300 W Xe lamp. In the test, 0.1 g of the photocatalyst was added to 100 mL of the aqueous solution (containing 15% glycerol as a sacrificial agent), dispersed ultrasonically, and then magnetically using a magnetic stirrer. Before light illumination, the system was evacuated of any remaining air by a flow of N_2_ gas for 30 min, and then the light was switched on. The reaction time was measured for 5 h. The resulting hydrogen was analyzed by a TCD-type gas chromatograph (HP 5890A, Carboxen 1010 plot).

## 3 Results and Discussion

### 3.1 XRD Analysis

The XRD patterns of pure ZnO, Ag_2_O–ZnO, and CuO/Ag_2_O–ZnO heterostructures are shown in Figure 1. The XRD patterns of pure ZnO (Figure 1a) showed different peaks indexed to (100), (002), (101), and (110) planes of wurtzite ZnO, in agreement with the JCPDS card of ZnO (JCPDS 36-1451).^32,34,42^ In addition, Figure 1 illustrates all of the modified ZnO samples showing the same hexagonal wurtzite lattice structure. On the other hand, the XRD pattern of Ag_2_O–ZnO (Figure 1b) displays new peaks that appear at 2θ values of 33.11°, 37.91°, 44.24°, 55.01°, 65°, and 73.14° corresponding to the cubic structure of Ag_2_O nanoparticles (JSPDS no. 41-1104).^43,44^ Furthermore, the CuO/Ag_2_O–ZnO heterostructure (Figure 1c–e) showed a new peak that appeared at 35.44°, 38.11°, 61.65°, and 66.55° corresponding to crystal planes (002), (111), (202), and (11–3) of CuO nanoparticles, respectively, with the data provided in (JCPDS 00-048-1548).^19,45^ Also, the intensity of ZnO peaks was obviously reduced after the addition of Ag^+^ (Figure 1), while the addition of Cu^2+^ was accompanied by an increase in the peaks’ intensity. On the other hand, peaks shifting to the lower 2θ-angles are detected, as shown in Figure S1 (Supporting Information), after the addition of Ag^+^ and Cu^2+^, indicating the partial substitution of Zn^2+^ ions by Ag^+^ and Cu^2+^ in the ZnO lattice. Also, Figure S1 displays that the shift in the peaks’ position to lower 2θ values was large after the addition of Ag^+^ compared with the shifting that was detected after the addition of Cu^2+^. This resulted due to the difference in the ionic radius of Ag^+^ (1.26 Å) which was larger than that of Zn^2+^ (0.74 Å), leading to an obvious shift, while the ionic radius of Cu^2+^ (0.73 Å) was nearly similar to the ionic radius of Zn^2+^, leading to the easy substitution of Zn^2+^ by Cu^2+^ ions, and consequently, a slight shift in the peaks’ positions was detected.^35,39,46,47^ According to the above results, Ag^+^ and Cu^2+^ are successfully doped into the ZnO lattice.^45,48^ The crystallite sizes of the fabricated photocatalysts were calculated using the Debye–Scherrer equation. Table 1 illustrates that the addition of Ag_2_O was accompanied by a decrease in the crystal size of ZnO to 30.8 nm. However, the crystal size of Ag_2_O–ZnO increased after the addition of CuO. Furthermore, Table 1 demonstrates that the crystal size increased with increasing wt % of CuO.

**Figure 1:**
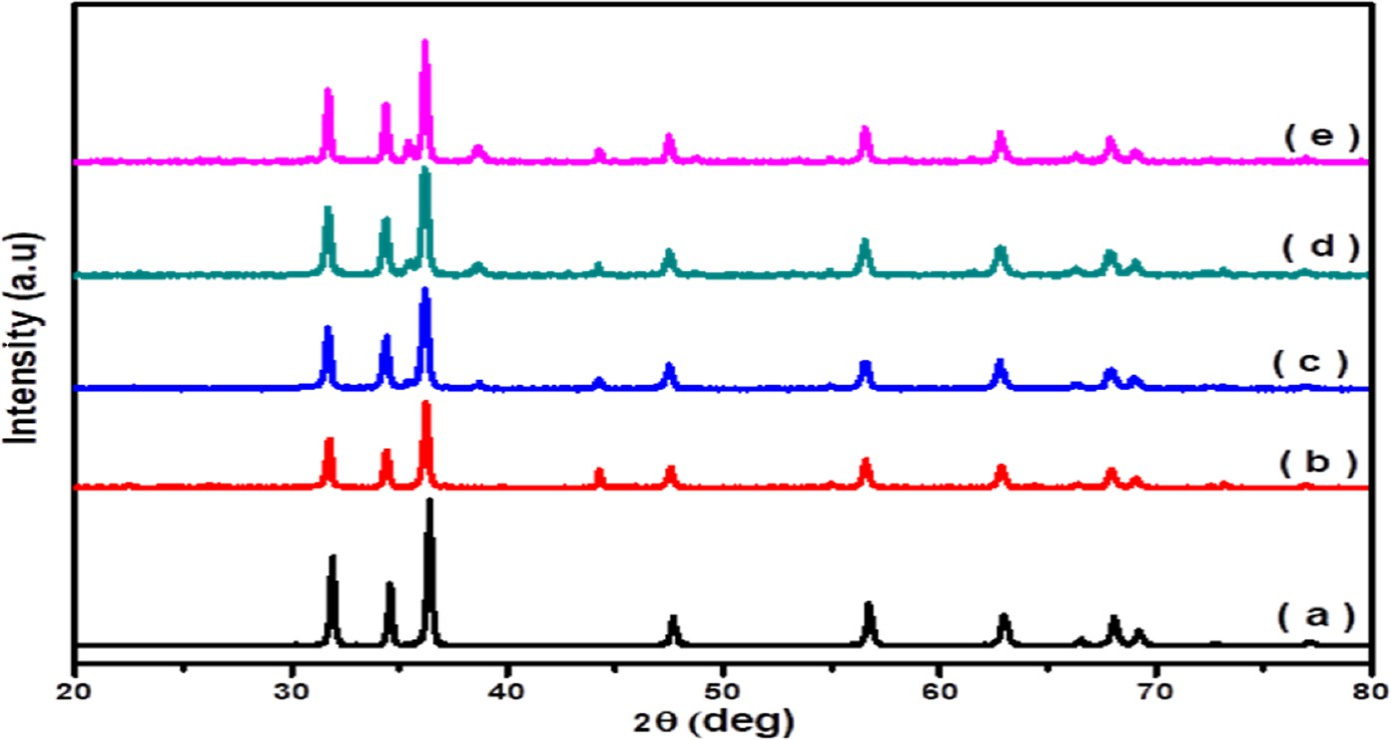
XRD spectra of (a) ZnO, (b) Ag_2_O–ZnO, (c) 3% CuO/Ag_2_O–ZnO, (d) 5% CuO/Ag_2_O–ZnO, and (e) 10% CuO/Ag_2_O–ZnO heterostructures.

**Table 1:** Crystal Size and Band Gap Size of the Prepared Photocatalysts

sample name	crystallite size D (nm)	band gap energy (eV)
ZnO	40.2	3.13
Ag_2_O–ZnO	30.8	2.95
3% CuO/Ag_2_O–ZnO	32.7	2.93
5% CuO/Ag_2_O–ZnO	32.5	2.60
10% CuO/Ag_2_O–ZnO	34.9	2.35

### 3.2 TEM Analysis

To further investigate the morphology of the fabricated photocatalysts, we analyzed them by TEM. As displayed in Figure 2, the TEM image of pure ZnO (Figure 2a) showed a hexagon-like shape with different particle sizes varying in the range between 17 and 60 nm. Figure 2b shows the typical TEM image of Ag_2_O–ZnO, which displays the effect of Ag_2_O addition on the morphology of ZnO. The image shows hexagonal and spherical shapes where Ag_2_O appears as spherical particles attached to the surface of ZnO. On the other hand, the image (Figure 2b) shows that the particle size decreased after the addition of Ag_2_O compared with pure ZnO. TEM images in Figure 2c,d illustrate the effect of the addition of CuO on the morphology of Ag_2_O–ZnO nanoparticles, where the images demonstrate that the samples have hexagonal and spherical shapes, and the particles of CuO appear on the surface of ZnO. In addition, the images (Figure 2c,d) show that the particle size of the CuO/Ag_2_O–ZnO heterostructure decreased compared with ZnO and Ag_2_O–ZnO, which conforms to the XRD results.

**Figure 2:**
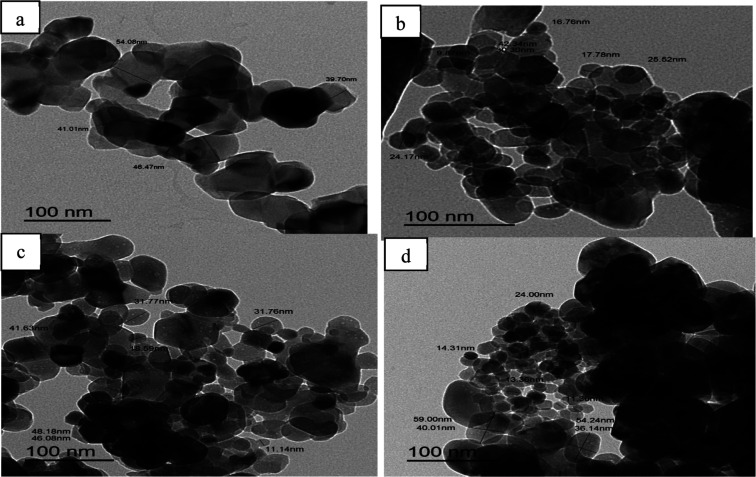
TEM images of (a) ZnO, (b) Ag_2_O–ZnO, (c) 5% CuO/Ag_2_O–ZnO, and (d) 10% CuO/Ag_2_O–ZnO heterostructures.

### 3.3 SEM and EDS with Elemental Mapping

The surface morphology of 5% CuO/Ag_2_O–ZnO was investigated using SEM analysis. The SEM image (Figure 3) displays that the 5% CuO/Ag_2_O–ZnO heterostructure is composed of aggregated particles with different sizes and shapes. In addition, the SEM image shows aggregated particles on the surface of ZnO, which confirms the XRD results.

**Figure 3:**
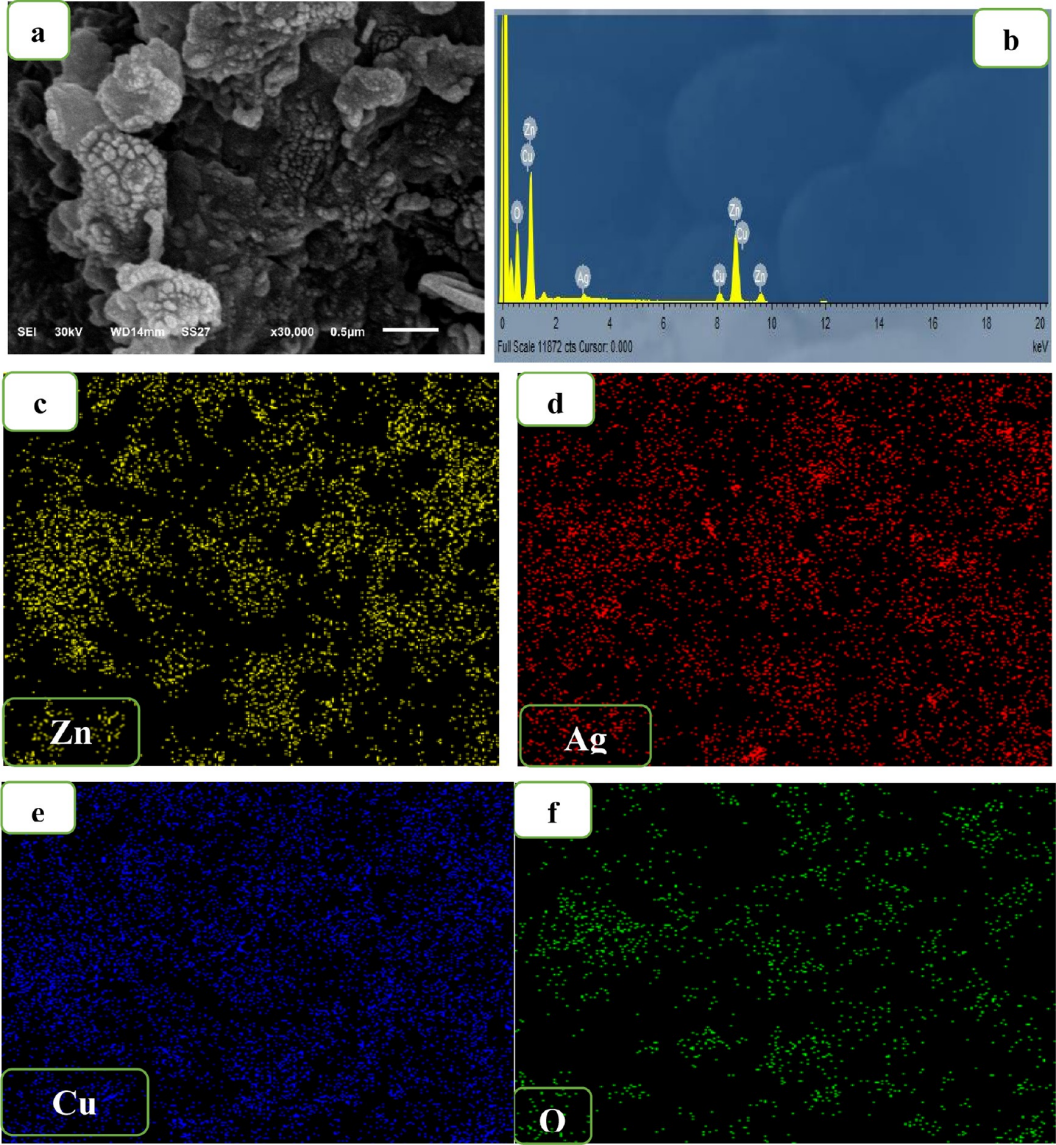
SEM image (a), EDS spectrum (b), and elemental mappings: ((c) Zn, (d) Ag, (e) Cu, and (f) O) of the 5% CuO/Ag_2_O–ZnO heterostructure.

Energy-dispersive X-ray spectroscopy (EDS) and the elemental mapping analysis were carried out to further confirm the composition and the homogeneous distribution of CuO, Ag_2_O, and ZnO in the 5% CuO/Ag_2_O–ZnO heterostructure. The EDS spectrum (Figure 3b) confirms the presence of Zn, Cu, Ag, and O in the composition of 5% CuO/Ag_2_O–ZnO. The elemental mapping analysis (Figure 3c–f) shows a good distribution of Zn, Cu, Ag, and O in the 5% CuO/Ag_2_O–ZnO heterostructure, which confirms again that the addition of CuO and Ag_2_O into ZnO is successful and uniform.

### 3.4 XPS Analysis

The surface chemical composition and oxidation states of the fabricated photocatalysts were further examined by the XPS technique. Figure 4a shows the survey spectrum of the 5% CuO/Ag_2_O–ZnO heterostructure and reveals that the sample is composed of Zn, Ag, Cu, and O elements. The high-resolution XPS spectrum of Zn 2p (Figure 4b) displays two peaks located at 1022.35 and 1045.55 eV, which are assigned to Zn 2p3/2 and Zn 2p1/2, respectively, indicating the formation of Zn^2+^. The XPS spectrum of Cu 2p (Figure 4c) shows two main peaks with binding energies at 933 eV, conjugated with a satellite peak located at 943 eV and the second peak located at 953.18 eV, with a satellite peak at 962 eV corresponding to Cu 2p3/2 and Cu 2p1/2, respectively. It is worth noting that the presence of satellite peaks at higher binding energy is attributed to the formation of Cu^2+^, which is associated with the existence of Cu 2p^5^3d^9^ electronic configuration.^49–51^ However, no satellite peaks are exhibited in the case of d^10^ electron configuration that is attributed to Cu^0^ and Cu^1+^.^51,52^ Figure 4d shows the Ag 3d XPS spectrum, in which two peaks are seen at 367.46 and 373.45 eV assigned to Ag 3d5/2 and Ag 3d3/4, respectively, with a spin–orbital splitting of photoelectrons at 5.98 eV corresponding to oxidized Ag_2_O.^53,54^ The XPS spectrum of O 1s (Figure 4d) consists of three peaks located at 530.35 eV belongs to O^2–^ ions in the wurtzite ZnO structure, and 531.79 eV belongs to Cu–O–Zn and Ag–O–ZnO, while the peak at 532.24 eV is attributed to the oxygen adsorbed on the photocatalyst surface.^55^

**Figure 4:**
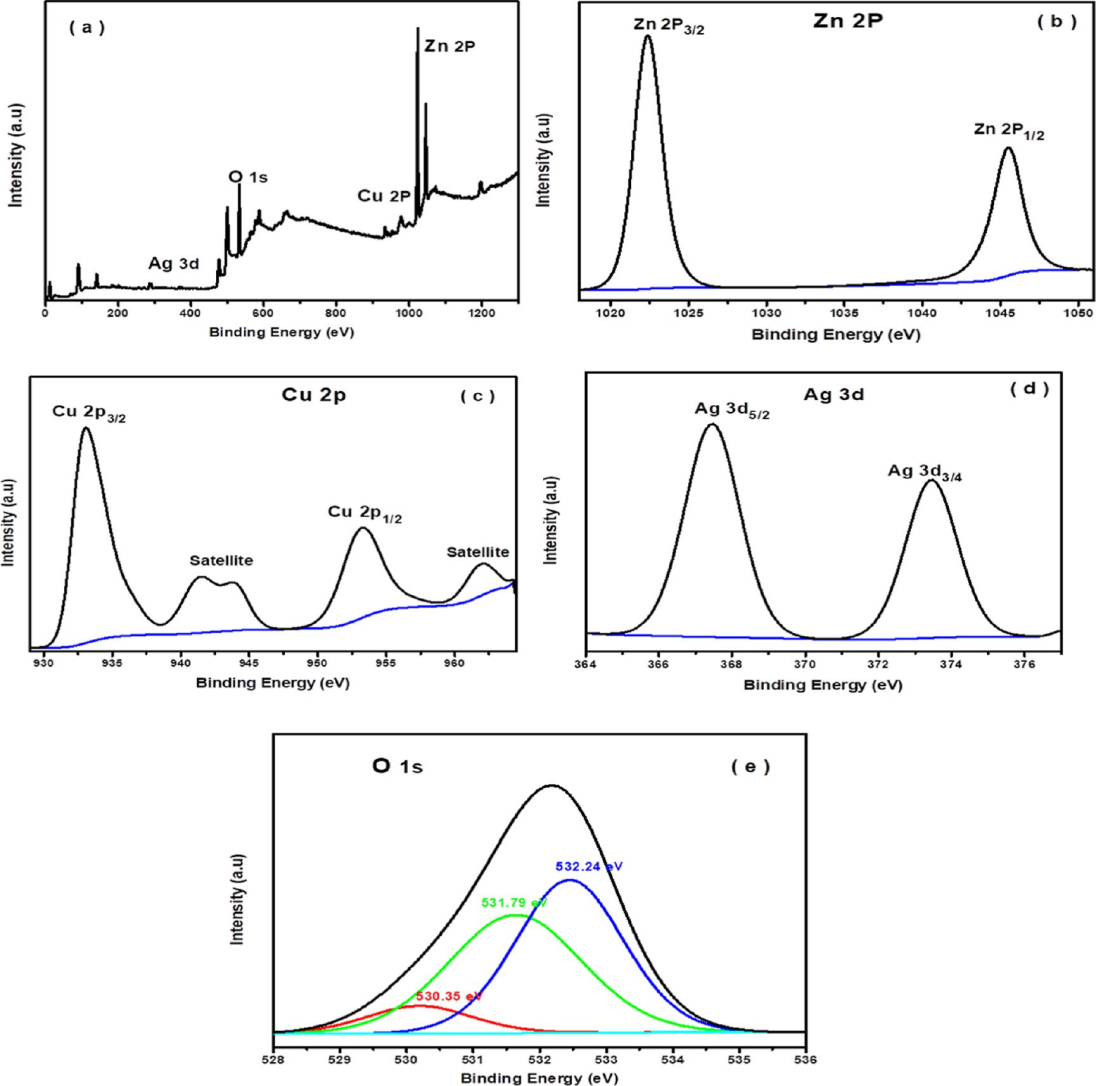
XPS survey spectra (a) and high-resolution spectra for (b) Zn 2p, (c) Cu 2p, (d) Ag 3d, and (e) O 1s of the 5% CuO/Ag_2_O–ZnO heterostructure.

### 3.5 FTIR Spectroscopy Analysis

Figure 5 shows the FTIR spectra of the pure and modified ZnO nanoparticles. The spectra displayed wide bands located at 3450 cm^–1^ and two other bands at 1620 cm^–1^ and 1370 cm^–1^ for both pure and doped ZnO, assigned to the −OH stretching vibrations and/or adsorbed H_2_O.^56,57^ The FTIR spectrum of pure ZnO (Figure 5a) exhibits absorption bands at 645–466 cm^–1^ attributed to the vibrational modes of the Zn–O bond.^58,59^ In addition, ZnO shows absorption bands around 921 and 986 cm^–1^, which may be related to the stretching vibration of O–Zn–O.^60–62^ The FTIR spectrum of Ag_2_O–ZnO (Figure 5b) shows absorption bands at 473 and 845 cm^–1^ ascribed to Ag–O, while the band at 1036 cm^–1^ is attributed to Ag–O–Zn.^63^ On the other hand, the FTIR spectra of the CuO/Ag_2_O–ZnO heterostructure (Figure 5c–e) show new absorption bands located at 874, 762, 560, 488, and 450 cm^–1^, which are assigned to Cu–O.^7,64^ In addition, another band appeared at 1123 cm^–1^ that was attributed to Cu–O–Zn. It is worth noting that some of the absorption bands were shifted to the higher frequency, indicating the substitution of Ag^+^ and Cu^2+^ ions into the Zn–O lattice.^65^

**Figure 5:**
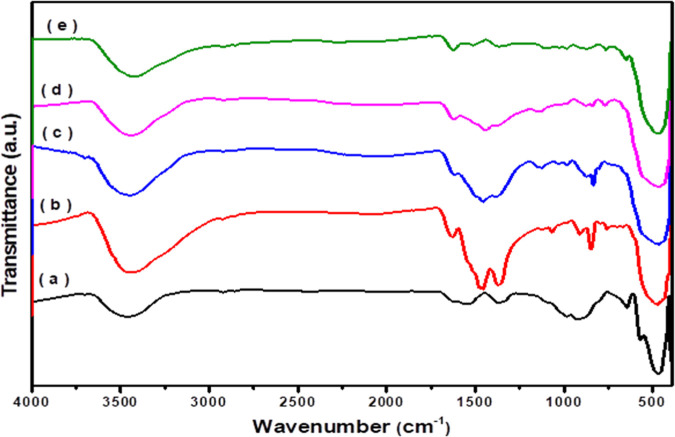
FTIR spectra of (a) ZnO, (b) Ag_2_O–ZnO, (c) 3% CuO/Ag_2_O–ZnO, (d) 5% CuO/Ag_2_O–ZnO, and (e) 10% CuO/Ag_2_O–ZnO heterostructures.

### 3.6 UV–Vis Spectroscopy Analysis

The optical characteristics of the fabricated photocatalysts were studied. Figure 6 shows the UV–vis absorption spectra of pristine and doped ZnO. The spectrum of pristine ZnO shows absorption bands under 400 nm (Figure 6a) due to the electronic interband transitions.^66,67^ The addition of Ag^1+^ and CuO led to a significant enhancement in the visible absorption, and a large red shift of the band edges absorption was detected, indicating that the doping of ZnO with Ag_2_O and CuO led to formation of additional absorption bands in ZnO. On the other hand, the shift in the absorption edge to higher wavelengths increased gradually with increasing CuO concentration. Furthermore, the addition of CuO led to appearance of an additional broad absorption band at 930–960 nm, which indicates the partial substitution of Cu^2+^ ions into the ZnO lattice.^68^ Additionally, the intensity and breadth of the band increased with increasing wt % of CuO content, indicating that the addition of CuO enhanced the absorption of ZnO in the visible region. The optical band gap (*E*_g_) was calculated using Tauc’s equation as displayed in Figure S2. The obtained values of *E*_g_ are listed in Table 1. From Table 1, the *E*_g_ of ZnO is 3.13 eV, while the *E*_g_ of doped ZnO reduced gradually with increasing CuO concentration. The enhancement of absorption in the visible region and narrowing of the band gap of ZnO after the addition of Ag_2_O and CuO is attributed to the formation of heterojunctions, where Ag_2_O acts as a photosensitizer, which improves the absorption of visible light.^53^ The addition of Ag_2_O and CuO into ZnO leads to the sp-d spin (s-d and p-d) exchange interactions between the localized electrons of Ag_2_O and CuO and band electrons of ZnO, which in turn give correction of VB and CB edge positions and consequently narrow the *E*g.^69^

**Figure 6:**
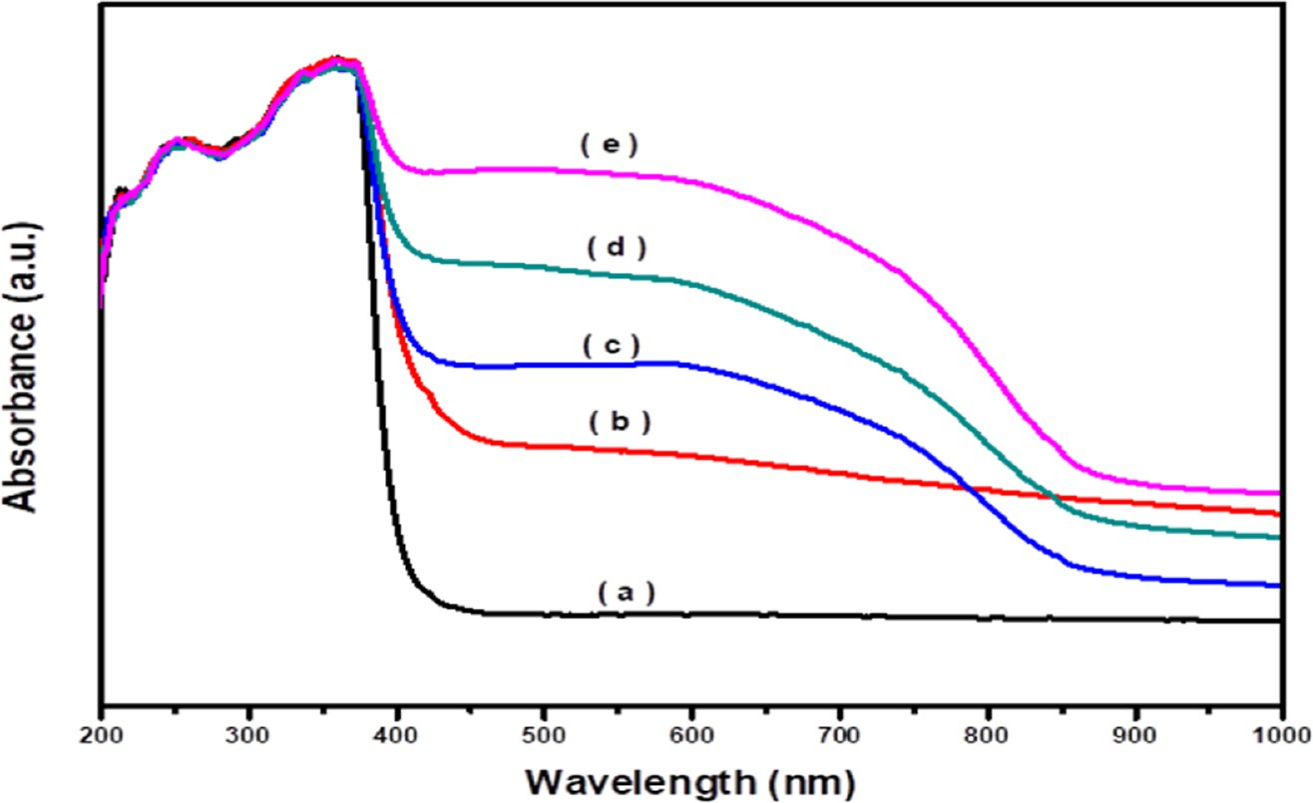
UV–vis spectra of (a) ZnO, (b) Ag_2_O–ZnO, (c) 3% CuO/Ag_2_O–ZnO, (d) 5% CuO/Ag_2_O–ZnO, and (e) 10% CuO/Ag_2_O–ZnO heterostructures.

### 3.7 PL Analysis

To investigate the efficiency of Ag_2_O and CuO in enhancing the separation and retarding the recombination of photon-generated e^–^/h + pairs of ZnO, PL analysis of pure and doped ZnO was performed, as shown in Figure 7. Pure ZnO shows high PL intensity compared with other samples, indicating the high and fast recombination of charge carriers due to ZnO having a wide band gap. However, the addition of Ag_2_O and CuO was accompanied by a remarkable decrease in the PL intensity, indicating that Ag_2_O and CuO played an effective role in retarding or restricting the recombination of photogenerated electron and hole pairs, prolonging their lifetime and thereby enhancing the photocatalytic activity of doped ZnO. Furthermore, Figure 7 illustrates that the PL intensity emissions decreased remarkably with increasing CuO concentration, and the 5% CuO/Ag_2_O–ZnO heterostructure showed the lowest intensity, indicating that the addition of 5 wt % of CuO is the optimal concentration for inhibiting the recombination of e^–^/h + pairs effectively. On the other hand, increasing the concentration of CuO up to 10 wt % was accompanied by an increase in the PL intensity, confirming that the presence of a high concentration of CuO played a reverse role as a recombination center. Increasing the concentration of CuO leads to an increase in the number of Cu^2+^ ions that are introduced into the ZnO lattice, which in turn lowers the band bending on the crystallite surface of the photocatalyst and consequently decreases the driving force that acts on the photogenerated e^–^/h^+^ pairs, leading to a reduction in the separation rate of e^–^/h^+^ pairs.^26,70^ According to the above results, the addition of Ag_2_O and CuO effectively improved the separation and accelerated the transfer of e^–^/h^+^, indicating the design of CuO/Ag_2_O–ZnO heterostructure strongly improved the generated photoinduced e^–^/h^+^ pairs and minimized their recombination effectively.^32^

**Figure 7:**
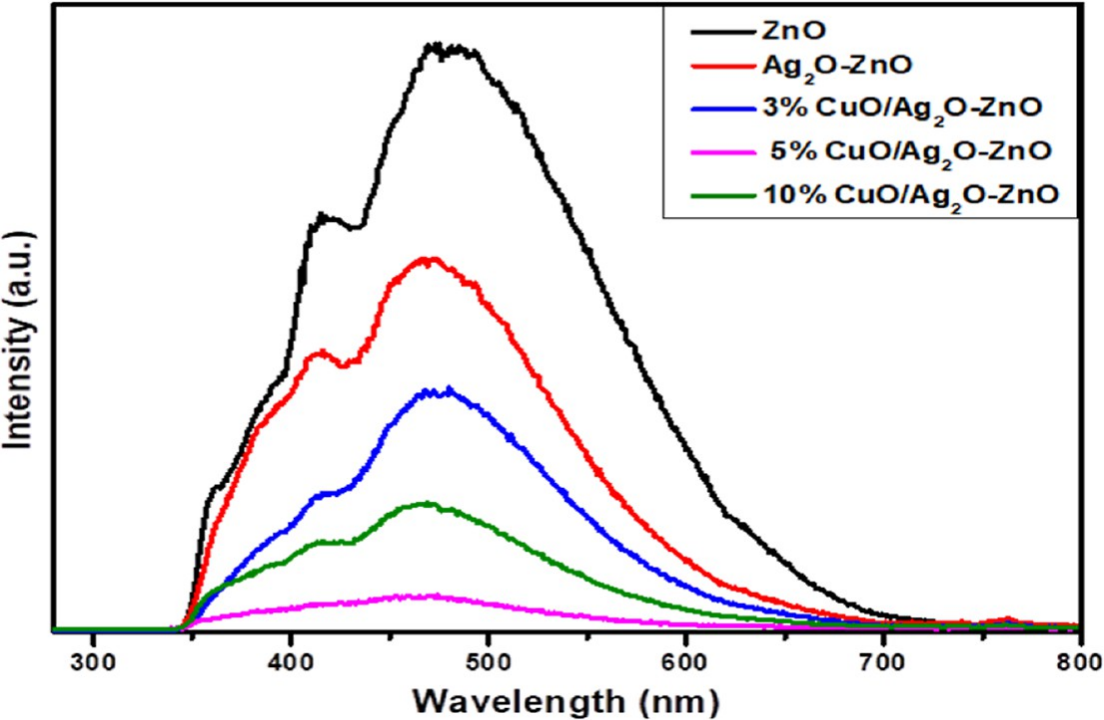
PL spectra of ZnO and Ag_2_O–ZnO and CuO/Ag_2_O–ZnO heterostructures with different CuO contents.

### 3.8 Photocatalytic Activity

#### 3.8.1 Photodegradation of MB and TC

The photocatalytic performance of ZnO, Ag_2_O–ZnO, and CuO/Ag_2_O–ZnO heterostructures with different CuO amounts was evaluated by photodegradation of MB and TC under natural sunlight, as shown in Figure 8. No degradation of MB and TC was detected in the absence of photocatalysts or under an illumination source, which ruled out the possibility of self-degradation. Figure 8 depicts that the photodegradation efficiency of pure ZnO is enhanced sharply after the addition of Ag_2_O and CuO. Additionally, the photodegradation of MB and TC improved obviously with increasing concentration of CuO, and the 5% CuO/Ag_2_O–ZnO heterostructure showed the highest photodegradation efficiency compared to other contents, with nearly 100% after 60 min in the case of MB and 80 min for TC. These results illustrate that the addition of Ag_2_O and CuO played an important role in reducing the *E*_g_ of ZnO and significantly improved the absorption in the visible region. Also, the addition of CuO enhanced the separation and inhibited the recombination of e^–^/h^+^ pairs effectively by the creation of a *p*–*n* heterojunction. On the other hand, increasing the concentration of CuO up to 10 wt % accompanied by reduction in the photodegradation of MB and TC indicated that CuO at high concentrations acted as a recombination center of photogenerated e^–^/h^+^, and also, the number of the active sites on the surface of ZnO became unavailable due to the aggregation of CuO on the surface of ZnO, as observed in XRD results.

**Figure 8:**
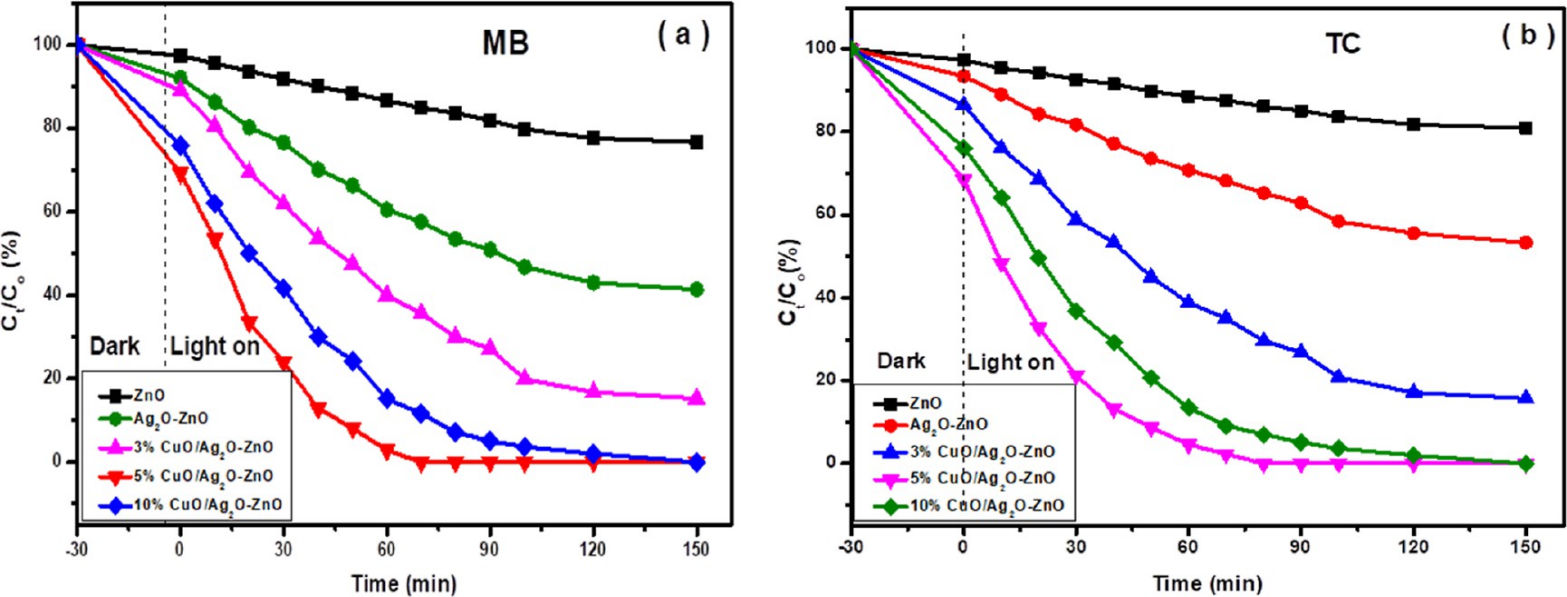
Photodegradation performance of (a) MB and (b) TC over pure and doped ZnO nanoparticles.

For comparison, the photocatalytic performance of CuO/Ag_2_O nanoparticles was investigated by photodegradation of MB and TC, as shown in Figure S3. The binary 5% CuO/Ag_2_O nanoparticles showed low photodegradation efficiency compared with the ternary 5% CuO/Ag_2_O–ZnO, with nearly 70% after 60 min in the case of MB and 65% for TC. According to these results, the low photocatalytic performance of 5% CuO/Ag_2_O resulted from the high recombination rate, which limited the number of photogenerated charges, leading to a reduction in the photocatalytic activity of 5% CuO/Ag_2_O compared with 5% CuO/Ag_2_O–ZnO. The presence of ZnO plays an important role in reducing or preventing the recombination rate of photogenerated charges, leading to an enhancement of the photodegradation rate for MB and TC.

##### 3.8.1.1 Effect of pH

The effect of pH on the photodegradation of MB and TC over 5% CuO/Ag_2_O–ZnO heterostructure was investigated at pH = 2, 4, 6, 8, 10, and 12 under the same conditions of dye concentration and weight of the photocatalyst. Figure S4 shows that the photodegradation of MB increased as the pH increased from 2 to 10, indicating that the basic conditions are optimal for the photodegradation of MB. On the other hand, Figure S4 displays that the photodegradation of TC increased upon increasing the pH from 2 to 8 and then decreased with increasing pH values. The highest photodegradation of MB and TC was obtained at pH 10 and 8, respectively. The behavior of MB and TC in the acidic and basic media can be explained on the basis of the point-of-zero charge (pH_ZPC_) of the CuO/Ag_2_O–ZnO photocatalyst, which is determined to be 8.3, as shown in Figure S5. At pH > pH_pzc_ (acidic medium), the surface of the photocatalyst is positively charged, whereas at pH < pH_pzc_ (basic medium), the surface is negatively charged. Consequently, in an acidic medium, both the surface of the photocatalyst and MB molecules are positively charged, leading to electrostatic repulsion between them, which results in lower photodegradation of MB. However, in the basic medium (pH < pH_pzc_), the surface of the photocatalyst is negative and MB is positive, leading to an enhancement in the photodegradation of MB due to the electrostatic attraction between them. It was reported that the TC molecules are positively charged at pH > 3.3 and become neutral zwitterions at pH = 3.3–7.7 and are negatively charged at pH < 7.7.^71,72^ However, the photodegradation of TC increases as the pH increases from 3 to 7 due to a change in the TC charge from positive to neutral, while the surface of the photocatalyst and TC becomes negative in the basic medium (pH < pH_pzc_), resulting in electrostatic attraction, which in turn decreases the photodegradation of TC.

##### 3.8.1.2 Kinetic Study

The photodegradation kinetics of MB and TC were studied by pseudo-zero-order, Hinshelwood pseudo-first-order, and pseudo-second-order kinetic models according to eqs 2, 3, and 4, respectively

2 $$C_{o}-C_{t}=kt$$

3 $$\ln\left(\frac{C_{o}}{C_{t}}\right)=kt$$

4 $$\frac{1}{C_{t}}-\frac{1}{C_{o}}=kt$$

where *k* and *t* are the time and photodegradation rate constant. Figures S6–S8 depict the obtained kinetic curves of photodegradation of MB and TC from pseudo-zero-order, pseudo-first-order, and pseudo-second-order kinetic models, respectively. The calculated parameters are listed in Tables S1, 2, and S3, respectively. According to the high values of correlation coefficients *R* ^2^ and excellent fitness, the photodegradation of both MB and TC follows the Hinshelwood first-order kinetic model. In addition, the 5% CuO/Ag_2_O–ZnO heterostructure exhibited the highest degradation rate constant compared with other samples, being 0.0436 mg^–1^·L·min^–1^ and 0.0431 mg^–1^·L·min^–1^ for MB and TC, respectively. The ternary (three-component) photocatalysts showed high photocatalytic activity, which may be due to the formation of heterojunctions p–n between the three components and the synergistic effect between them.

**Table 2:** Kinetic Parameters of the First-Order Model for MB and TC Photodegradation

samples	MB		TC	
	K_1_ (mg^–1^·L·min^–1^)	*R* ^2^	K_1_ (mg^–1^·L·min^–1^)	*R* ^2^
ZnO	0.0021	0.9986	0.0018	0.9931
Ag_2_O–ZnO	0.0071	0.9988	0.0051	0.9968
3% CuO/Ag_2_O–ZnO	0.0126	0.9983	0.0132	0.9993
5% CuO/Ag_2_O–ZnO	0.0436	0.9786	0.0431	0.9933
10% CuO/Ag_2_O–ZnO	0.0247	0.9938	0.0275	0.9922

The mineralization of MB and TC over the 5% CuO/Ag_2_O–ZnO heterostructure was investigated using the total organic carbon (TOC %) removal method, and the obtained results are shown in Figure 9. The mineralization of MB and TC increased with increasing photodegradation time, and the TOC % of MB and TC was 85.3% and 75.8% after 180 min, respectively. However, the TOC % of both MB and TC became 100% after 240 min of photodegradation reaction, indicating the complete mineralization of MB and TC.

**Figure 9:**
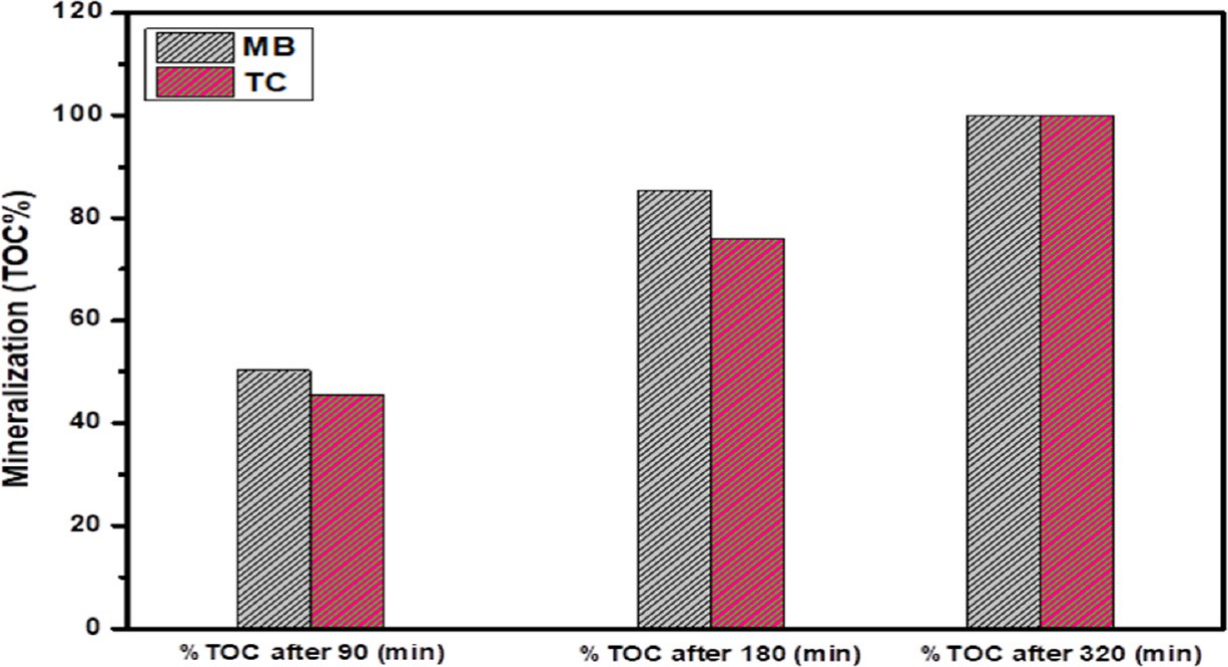
% TOC removal of MB and TC vs time over the 5% CuO/Ag_2_O–ZnO heterostructure.

Table S3 illustrates a comparison of the photocatalytic efficiency between the photocatalysts prepared in this study and the photocatalysts previously described in the literature. The results revealed that the prepared photocatalysts in this work have excellent stability, reusability, and photoactivity compared with other chosen literature.

#### 3.8.2 Hydrogen Production

The photocatalytic activity of pure and doped ZnO was investigated by hydrogen production from water splitting under sunlight simulation in the presence of glycerol as a hole scavenger. Figure 10 depicts the H_2_ evolution efficiency for ZnO, which is 50 μmol/g, while the doped samples show a significant increase in H_2_ production compared with pure ZnO. Furthermore, the production of H_2_ increased with increasing CuO concentration, and the sample with the 5% CuO/Ag_2_O–ZnO heterostructure showed the highest hydrogen production of 6092 μmol/g. The remarkable enhancement in the production of hydrogen in doped samples is attributed to the role of Ag_2_O and CuO in enhancing the separation and reducing recombination of charge carriers and consequently enhancing the photocatalytic activity of ZnO.

**Figure 10:**
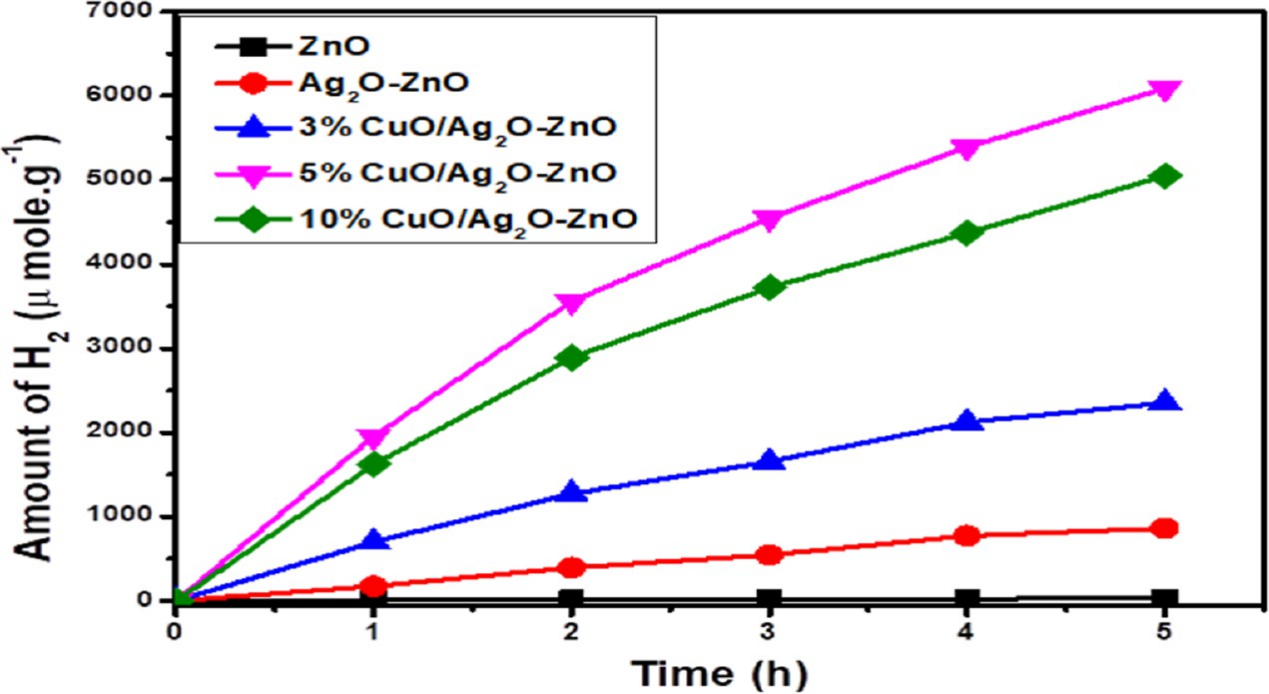
Amount of H_2_ production over the pure and doped ZnO heterostructure.

#### 3.8.3 Photodegradation Mechanism

The photodegradation mechanism of MB and TC over pure and doped ZnO nanoparticles was investigated by adding different scavengers to determine the main reactive species that are responsible for photodegradation of MB and TC, as shown in Figure 11. It is obvious that the photodegradation of MB and TC without scavenger agents was 100%, while after the addition of BQ, ETA-Na, and IPA, the photodegradation rate of MB and TC was apparently inhibited. Also, Figure 10 reveals an obvious reduction in the photodegradation of MB and TC with the addition of IPA and BQ, indicating that ^•^OH and ^•^O_2_^–^ are the reactive species in the photodegradation of MB and TC, and especially, ^•^OH plays the most critical role.

**Figure 11:**
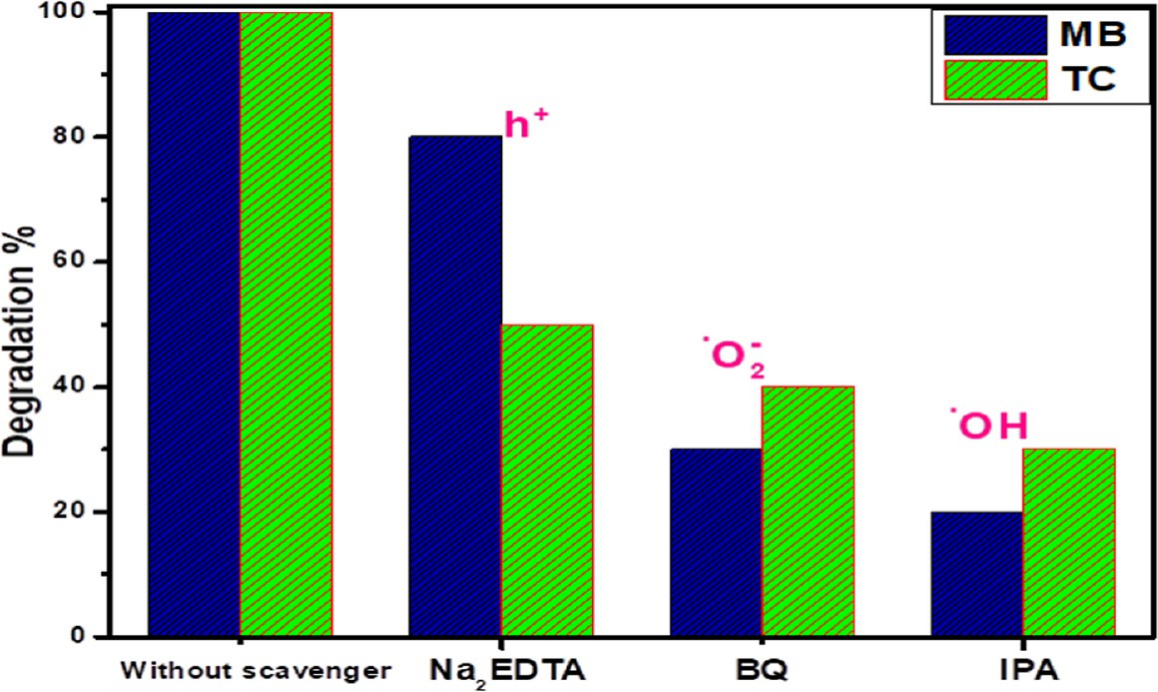
Effect of scavenger’s addition on the photodegradation of MB and TC over the 5% CuO/Ag_2_O–ZnO heterostructure.

The ESR measurement was also performed on the 5% CuO/Ag_2_O–ZnO heterostructure to further investigate the resulting active species during the photodegradation process, and DMPO was used as an active species-trapping agent. As shown in Figure S9, no signal peaks of DMPO– ^•^O_2_^–^ and DMPO– ^•^OH were detected under dark conditions. However, under light illumination, Figure S9 displays single peaks of DMPO– ^•^O_2_^–^ and DMPO– ^•^OH with relative intensity ratios of 1:1:1:1 and 1:2:2:1, respectively. These results confirm that ^•^O_2_^–^ and ^•^OH are the active radicals in the photodegradation of MB and TC.

The flat potential bands of ZnO, Ag_2_O, and CuO were calculated by a Mott–Schottky diagram. In Figure 12, ZnO shows a positive slope (Figure 12a), confirming that ZnO is an n-type semiconductor, while Ag_2_O and CuO display negative slopes of the linear plots (Figure 12b,c), indicating that both are p-type semiconductor materials. The flat-band potential (*E*_fb_) of ZnO, CuO, and Ag_2_O was measured to be −0.43, 1.10, and 0.65 V vs Ag/AgCl, which equals −0.23, 1.30, and 0.85 V vs NHE, respectively, according to the relation *E*_NHE_ = *E*_Ag/AgCl_ + *E*_oAg/AgCl_.^32^ According to the relationship between *E*_FB_, *E*_CB_, and *E*_VB_, in semiconductors, the difference between them was reported in the range of 0.1–0.2 V.^32^ Thus, the *E*_CB_ of ZnO and *E*_VB_ of CuO and Ag_2_O are determined to be −0.33, 1.20, and 0.75 V vs NHE, respectively. The band gap value of CuO and Ag_2_O was also determined to be 1.72 and 1.36 eV, respectively, as shown in Figure S10. On the other hand, the band edge positions of *E*_VB_ of ZnO and *E*_CB_ of CuO and Ag_2_O are calculated using the equation: *E*_VB_ = *E*_CB_ + *E*_g_, and the obtained values are 2.80 V of *E*_VB_ of ZnO and −0.52 V and −0.61 V of *E*_CB_ of CuO and Ag_2_O, respectively. As is known in heterojunction photocatalysts, the semiconductor that has a higher work function and a lower Fermi level (EF).^32^

**Figure 12:**
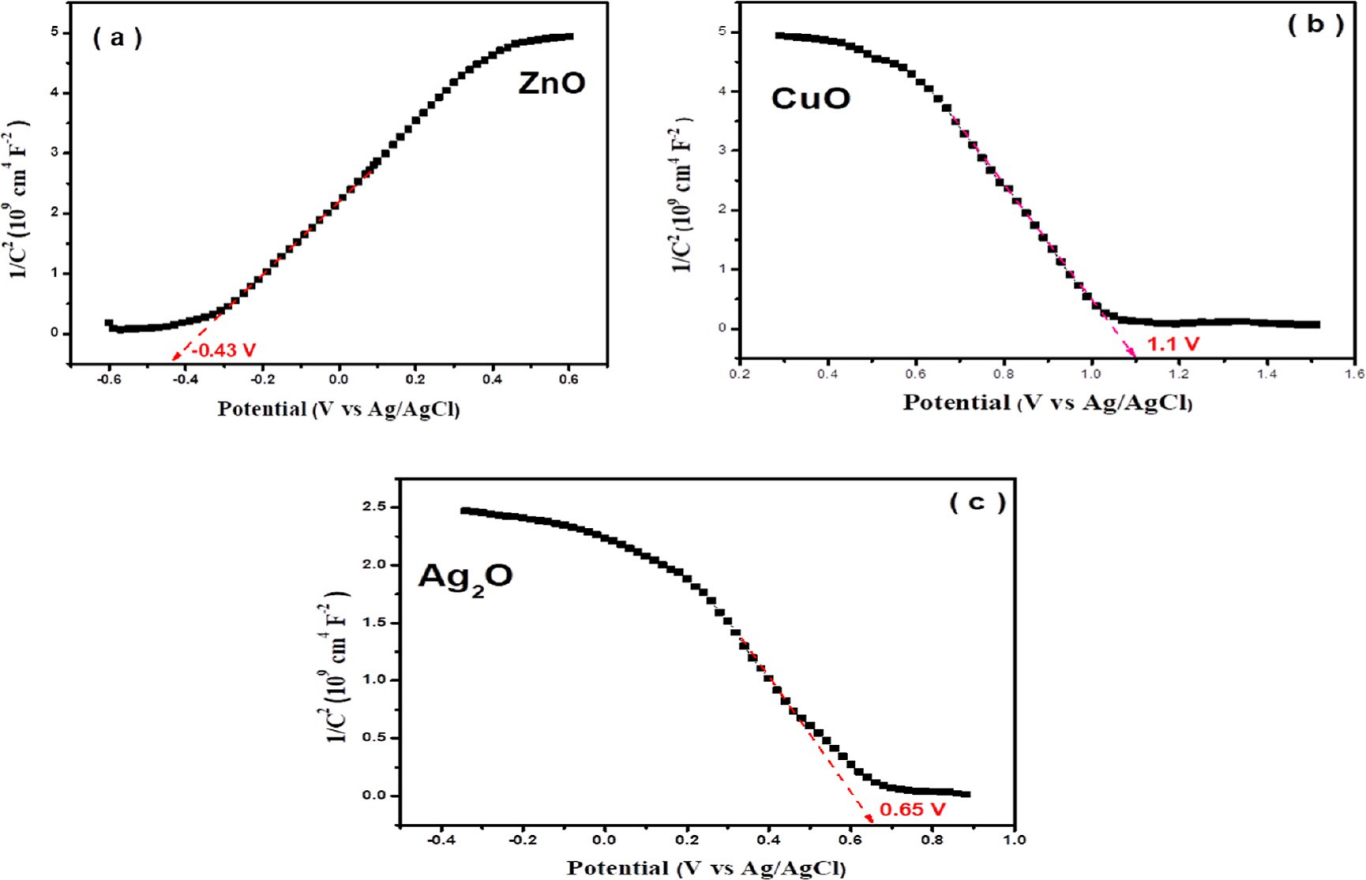
Mott–Schottky plots of (a) ZnO, (b) CuO, and (c) Ag_2_O.

Based on the obtained results, the S-scheme heterostructure mechanism (Figure 13) is the most appropriate to describe the photogeneration, transition, and separation of charge carriers, the photodegradation mechanism of MB and TC, and for H_2_ production over CuO/Ag_2_O–ZnO-heterojunction heterostructure photocatalysts. Upon contact of CuO and Ag_2_O to ZnO, intimate interfaces are formed and electrons transfer from CuO and Ag_2_O to ZnO, leading to the formation of an internal electric field (IEF) directed from CuO and Ag_2_O to ZnO, and consequently, electron depletion layers are formed at the interface of CuO, Ag_2_O, and ZnO due to accumulation of holes at the CuO and Ag_2_O side and electrons in the ZnO side.^33,51^ As a result, the Fermi level of CuO and Ag_2_O moved down, and that of ZnO moved up, while the band edges of CuO and Ag_2_O were bent upward, and those of ZnO were shifted downward.^32,51^

**Figure 13:**
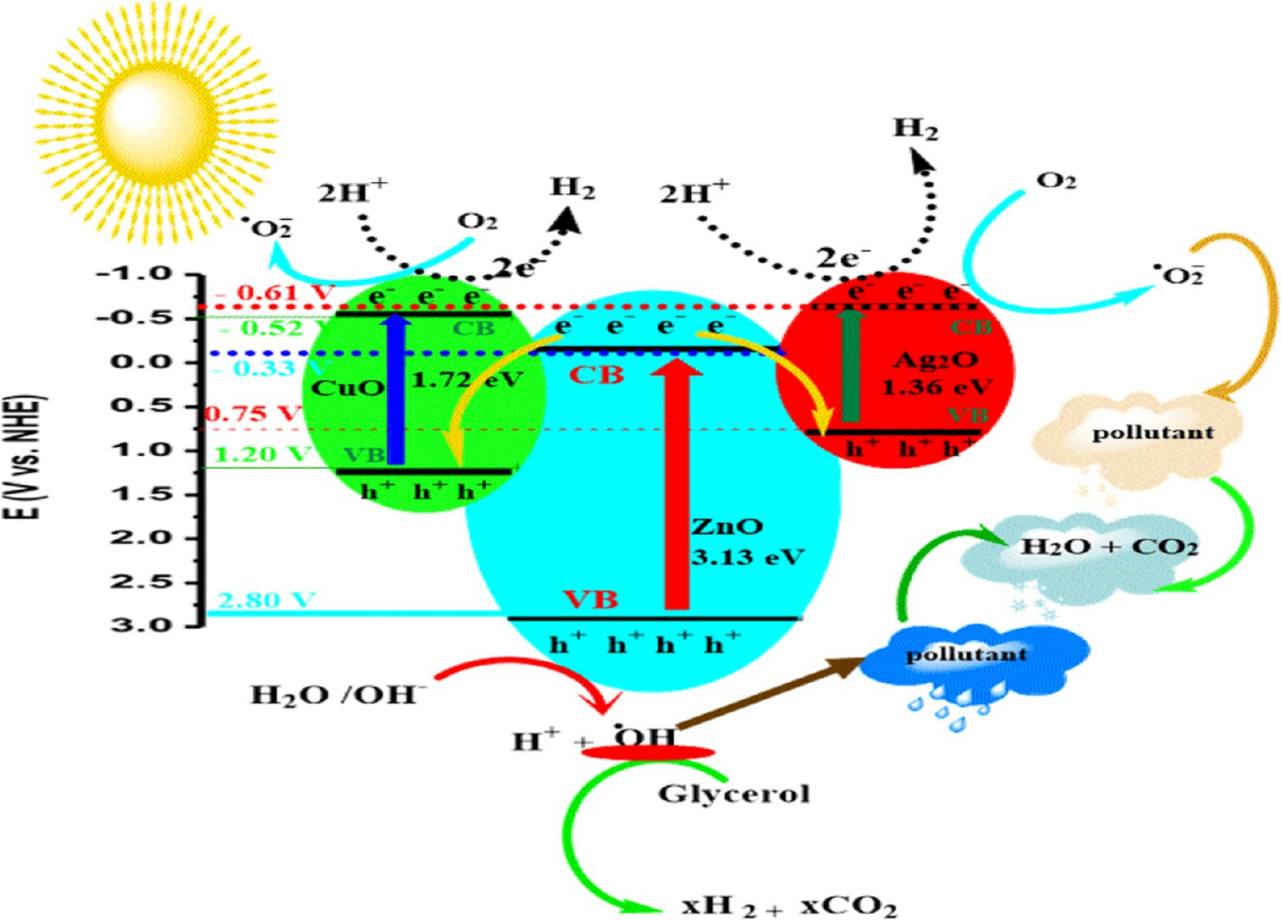
Schematic illustration of the S-scheme mechanism of the CuO/Ag_2_O–ZnO heterostructure.

When the CuO/Ag_2_O–ZnO heterostructure is illuminated under sunlight, both CuO, Ag_2_O, and ZnO are excited, and the photocarriers are produced at the CB and VB. However, the photogenerated electrons at the CB (e_CB_^–^) of ZnO are transferred at the interface of the CuO/Ag_2_O–ZnO heterostructure under the effect of IEF and band bending and recombined with photogenerated holes at the VB (h_VB_^+^) of CuO and Ag_2_O, while the photogenerated e_CB_^–^ at CuO and Ag_2_O and the photogenerated h_VB_^+^ at ZnO were retained and these photocarriers play the key role in the redox reactions. Moreover, the photogenerated e_CB_^–^ at the CB of CuO and Ag_2_O are strong reducing species, while the h_VB_^+^ at the VB of ZnO are strong oxidizing species. Consequently, the formation of a CuO/Ag_2_O–ZnO heterojunction heterostructure effectively enhanced the separation of photogenerated charges. On the other hand, the potential of the CB of CuO and Ag_2_O is more negative than that of O_2_/^•^O_2_^–^, indicating the electrons on e_CB_^–^ can reduce O_2_ to generate ^•^O_2_^–^. Furthermore, the photogenerated e_CB_^–^ at CuO and Ag_2_O has more negative potentials than that at 2H^+^/H_2_ and can reduce 2H^+^ to H_2_. On the contrary, the photogenerated h_VB_^+^ at ZnO can oxidize H_2_O and ^–^ OH to ^•^OH because the VB potential of ZnO is more positive than H_2_O/^•^OH and ^–^ OH/^•^OH.

#### 3.8.4 Recyclability and Stability

To inspect the photostability and reusability of the CuO/Ag_2_O–ZnO heterostructure, the sample was reused for the photodegradation of MB and TC for four repeated cycles under the same conditions. After the end of each cycle, the photocatalyst was recollected by centrifugation, washed with DI water and then ethanol, and finally dried at 80 °C for 8 h. Figure S11 reveals that the photodegradation of MB and TC was 94% and 92% after four runs, indicating a slight decrease in the photocatalytic efficiency of the CuO/Ag_2_O–ZnO heterostructure after the fourth run. On the other hand, the structural stability of the CuO/Ag_2_O–ZnO heterostructure after four runs was investigated by XRD analysis, and no considerable changes were detected, as displayed in Figure S12. Based on the above results, the CuO/Ag_2_O–ZnO heterostructure showed strong photoactivity and high stability and reusability, confirming the ability of these applied photocatalysts in wastewater treatment and hydrogen production.

The ion leaching from the photocatalytic degradation process was determined by inductively coupled plasma optical emission spectrometric (ICP-OES) analysis, and the obtained results displayed a very low amount of ions leaching from the prepared photocatalyst (>0.1%), indicating the good stability of the prepared photocatalyst during the photocatalytic reaction.

## 4 Conclusion

Active CuO/Ag_2_O–ZnO heterostructure photocatalysts were prepared by the sol–gel method. The influence of Ag^+^ and Cu^2+^ on the structural, optoelectronic, and photocatalytic properties of ZnO was investigated. XRD results confirmed the addition of Ag^+^ and Cu^2+^ into the lattice of ZnO. The optical analysis results displayed that the addition of Ag^+^ and Cu^2+^ into ZnO improved the visible light response, reduced the band gap energy, and enhanced the separation and inhibited the recombination of charge carriers of ZnO effectively. The sample with the 5% CuO/Ag_2_O–ZnO heterostructure showed the highest photocatalytic efficiency for photodegradation of MB, TC, and H_2_ evolution compared with other samples. Also, increasing the amount of CuO above 5 wt % was accompanied by a decrease in the photocatalytic performance of CuO/Ag_2_O–ZnO. The ESR analysis and radical quenching experiments demonstrated that the CuO/Ag_2_O–ZnO heterostructure can produce the •O_2_^–^ and •OH active radicals, which participate effectively in the photodegradation of MB and TC. The photodegradation kinetic results revealed that the photodegradation of MB and TC obeys the pseudo-first order model. The CuO/Ag_2_O–ZnO heterostructure showed superior photocatalytic activity under sunlight and excellent reusability and stability after four recycles, and it can be applied as a promising photocatalyst for wastewater treatment and H_2_ production.

## Supplementary Material



## Notes

XRD patterns of pure and doped ZnO nanoparticles; band gap energy of pure and doped ZnO nanoparticles; photodegradation performance of MB and TC over 5% CuO/Ag_2_O–ZnO and 5% Ag_2_O–ZnO nanoparticles; effect of reaction solution pH on photodegradation of MB and TC over the 5% CuO/Ag_2_O–ZnO heterostructure; determination of zero potential charge of the 5% CuO/Ag_2_O–ZnO heterostructure; linear plots of pseudo-zero-order, pseudo-first-order, and pseudo-second-order kinetic models of MB and TC photodegradation on the 5% CuO/Ag_2_O–ZnO heterostructure; ESR spectra of 5% CuO/Ag_2_O–ZnO heterostructure for ^•^OH and ^•^O^2–^ radicals; UV–vis spectra and band gap energy (inset) of CuO and Ag_2_O nanoparticles; effect of recycle times on the photocatalytic performance of the 5% CuO/Ag_2_O–ZnO heterostructure; effect of recycle on the structural properties of the 5% CuO/Ag_2_O–ZnO heterostructure; kinetic parameters of zero- and second-order models for MB and TC photodegradation; and comparison of the photodegradation efficiency of our photocatalysts with different photocatalysts reported in the literature (PDF)

## References

[1] Tahir M. B., Nabi G., Sagir M., Rafique M., Alrobei H., Nawaz T., Inayat A., Hussain S., Naz G., Iqbal K., Tahir M. S. (2021). Role of CTF in Bi_2_WO_6_/ZnO photocatalysts for effective degradation and hydrogen
energy evolution. Int. J. Hydrogen Energy.

[2] Oskoei A., Khaleghi M., Sheibani S. (2025). Modification
of MoS_2_/ZnO
nanocomposite for efficient photocatalytic degradation of water pollutants
and hydrogen evolution. J. Water Process Eng..

[3] Younas U., Mobeen F., Saleem A., Ali F., Huwayz M. A., Ashraf A., Ahmad A., Alwadai N., Pervaiz M., Iqbal M. (2024). Efficient hydrogen production via
overall water splitting using CuO/ZnO
decorated reduced graphene oxide as bifunctional electrocatalyst. Ceram. Int..

[4] Zang C., Han X., Chen H., Zhang H., Lei Y., Liu H., Wang C., Zhang G., Ge M. (2022). In situ growth of ZnO/Ag_2_O heterostructures on PVDF nanofibers as efficient visible-light-driven
photocatalysts. Ceram. Int..

[5] Hassan S. M., Ahmed A. I., Mannaa M. A. (2019). Preparation
and characterization
of SnO_2_ doped TiO_2_ nanoparticles: Effect of
phase changes on the photocatalytic and catalytic activity. J. Sci. Adv. Mater. Devices..

[6] Mannaa M. A., Qasim K. F., Alshorifi F. T., El-Bahy S. M., Salama R. S. (2021). Role of
NiO Nanoparticles in Enhancing Structure Properties of TiO_2_ and Its Applications in Photodegradation and Hydrogen Evolution. ACS Omega.

[7] Lone A. L., Rehman S. U., Haq S., Shahzad N., Al-Sadoon M. K., Shahzad M. I., Razzokov J., Shujaat S., Samad A. (2025). Unveiling
the physicochemical, photocatalytic, antibacterial and antioxidant
properties of MWCNT-modified Ag_2_O/CuO/ZnO nanocomposites. RSC Adv..

[8] Patwa R., Rohilla S., saini J., Goel N. (2025). Structural and spectroscopy
analysis of nanocomposites of metal oxide ZnO/CuO/Ag by coprecipitation:
Potential application in photocatalysis. Ceram.
Int..

[9] Luo K., Chen A., Liu Y., Yang J., Cai N., Li J. (2025). Constructing a highly
sensitive SERS sensor based on necklace-like
CNC/ZIF-8/Ag to detect and photo-degrade diquat in green tea leaves. Ind. Crops Prod..

[10] Parikirala R., Kore R., Rohini V., Venkateshwar Rao D., Chetti P., Pola S. (2024). Synthesis of new Cu/Zn
(II) complexes
for sonophotocatalysis for mineralization of pesticides and agrochemical
wastewater. J. Environ. Chem. Eng..

[11] Li X., Liu P., Li J. (2022). Magnetically separable Fe_3_O_4_/mZrO_2_/Ag nanocomposites: Fabrication and
photocatalytic activity. Colloids Surfaces A
Physicochem. Eng. Asp..

[12] V.R D., Subburu M., Gade R., Basude M., Chetti P., Simhachalam N. B., Nagababu P., Bhongiri Y., Pola S. (2021). A new Zn (ii)
complex-composite material: Piezo-enhanced photomineralization of
organic pollutants and wastewater from the lubricant industry. Environ. Sci. Water Res. Technol..

[13] Vallavoju R., Kore R., P R., Subburu M., Basude M., Chetti P., Pola S. (2023). Degradation of organic pollutants
in the presence of new Mn (II) complexes under ambient light or darkness
conditions. J. Photochem. Photobiol. A Chem..

[14] Subburu M., Gade R., Guguloth V., Chetti P., Ravulapelly K. R., Pola S. (2021). Effective photodegradation
of organic pollutantsin the presence of
mono and bi-metallic complexes under visible-light irradiation. J. Photochem. Photobiol. A Chem..

[15] Mannaa M. A., Mlahi M. R., AL Maofari A., Ahmed A. I., Hassan S. M. (2023). Synthesis
of Highly Efficient and Recyclable Bimetallic Co_x_-Fe_1‑x_-MOF for the Synthesis of Xanthan and Removal of
Toxic Pb^2+^ and Cd^2+^ Ions. ACS Omega.

[16] Mannaa M. A., Altass H. M., Salama R. S. (2021). MCM-41
grafted with citric acid:
The role of carboxylic groups in enhancing the synthesis of xanthenes
and removal of heavy metal ions. Environ. Nanotechnology,
Monit. Manag..

[17] Hassan S. M. H., Mannaa M. A., Ibrahim A. A. (2019). Nano-sized
mesoporous phosphated
tin oxide as an efficient solid acid catalyst. RSC Adv..

[18] Zhang S., Qu X., Lv H., Mao J., Zhou J. (2024). Hydrogen production
from low-temperature methanol steam reforming over silver-promoted
CuO/ZnO/Ag/Al_2_O_3_ Catalyst. Int. J. Hydrogen Energy.

[19] Meneceur S., Laouini S. E., Ali Mohammed H., Bouafia A., Salmi C., Ahmed Abdullah J. A., Alharthi F. (2025). Eco-Friendly ZnO/CuO/Ni Nanocomposites:
Enhanced photocatalytic dye adsorption and hydrogen evolution for
sustainable energy and water purification. J.
Cryst. Growth.

[20] Tahir M. B., Iqbal T., Kiran H., Hasan A. (2019). Insighting role of
reduced graphene oxide in BiVO 4 nanoparticles for improved photocatalytic
hydrogen evolution and dyes degradation. Int.
J. Energy Res..

[21] Guguloth V., Ahemed J., Subburu M., Guguloth V. C., Chetti P., Pola S. (2019). A very fast photodegradation
of dyes in the presence of new Schiff’s
base N4-macrocyclic Ag-doped Pd­(II) complexes under visible-light
irradiation. J. Photochem. Photobiol. A Chem..

[22] Ahemed J., Pasha J., Rao D V., Kore R., Gade R., Bhongiri Y., Chetti P., Pola S. (2021). Synthesis of new Zn
(II) complexes for photo decomposition of organic dye pollutants,
industrial wastewater and photo-oxidation of methyl arenes under visible-light. J. Photochem. Photobiol. A Chem..

[23] Alsulami Q. A., Rajeh A., Mannaa M. A., Albukhari S. M., Baamer D. F. (2021). Preparation of highly efficient sunlight driven photodegradation
of some organic pollutants and H2 evolution over rGO/FeVO_4_ nanocomposites. Int. J. Hydrogen Energy.

[24] Wen X., Liu Y., Zhang W., You L., Cai N., Li J. (2025). Recyclable
NiO/g-C_3_N_4_/ag hybrid substrates for sensitive
SERS detection and photo-degradation of residual pesticides in beverages. Food Chem..

[25] Zhang W., Chen M., Cheng X., Yang J., Li J. (2025). g-C3N4 modified
hollow Ag/ZrO_2_ core–shell composites for enhanced
solar photocatalytic degradation and recyclable SERS sensing of thiram
and methylene blue. Microchem. J..

[26] Gade R., Ahemed J., Yanapu K. L., Abate S. Y., Tao Y. T., Pola S. (2018). Photodegradation of organic dyes
and industrial wastewater in the
presence of layer-type perovskite materials under visible light irradiation. J. Environ. Chem. Eng..

[27] Vallavoju R., Kore R., Radhika P., Subburu M., Gade R., Basude M., Pola S., Chetti P. (2023). Enhanced piezo-photocatalytic
properties of new salophen based Ti (IV) complexes. Inorg. Chem. Commun..

[28] Masula K., Sreedhar P., Vijay Kumar P., Bhongiri Y., Pola S., Basude M. (2023). Synthesis and characterization
of NiO–Bi_2_O_3_ nanocomposite material for
effective photodegradation
of the dyes and agricultural soil pollutants. Mater. Sci. Semicond. Process..

[29] Abutalib M. M., Alghamdi H. M., Rajeh A., Nur O., Hezmad A. M., Mannaa M. A. (2022). Preparation of rGO/FeMoO4 as high-performance
photocatalyst
for degradation of malachite green, phenol and H2 evolution under
natural sunlight. Int. J. Hydrogen Energy.

[30] Alsulami Q. A., Rajeh A., Mannaa M. A., Albukhari S. M., Baamer D. F. (2022). One-step preparation of RGO/Fe_3_O_4_–FeVO_4_ nanocomposites as highly
effective photocatalysts
under natural sunlight illumination. Sci. Rep..

[31] Masula K., Bhongiri Y., Raghav Rao G., Vijay Kumar P., Pola S., Basude M. (2022). Evolution of photocatalytic
activity
of CeO_2_–Bi_2_O_3_ composite material
for wastewater degradation under visible-light irradiation. Opt. Mater. (Amst)..

[32] Ahmad I., Shukrullah S., Naz M. Y., Bhatti H. N., Khalid N. R., Ullah S. (2023). Rational design
of ZnO–CuO–Au S-scheme heterojunctions
for photocatalytic hydrogen production under visible light. Int. J. Hydrogen Energy.

[33] Abutalib M. M., Alghamdi H. M., Rajeh A., Nur O., Hezma A. M., Mannaa M. A. (2022). Fe_3_O_4_/Co_3_O_4_-TiO_2_ S-scheme photocatalyst for degradation of organic
pollutants and H2production under natural sunlight. J. Mater. Res. Technol..

[34] Gultom N. S., Bintang F. I., Zeleke M. A., Hutama A. S., Kuo D.-H., Horprathum M., Panatarani C., Faizal F., Adiperdana B. (2024). Optimizing
Antibacterial Photodynamic Theraphy of biocompatible ZnO/Ag_2_O p-n Heterojunction: In Vitro and In Silico study. Surfaces and Interfaces.

[35] Mejia-Bernal J. R., Gómez-Solís C., Juárez-Ramírez I., Ortiz-Rabell G., Díaz-Torres L.
A. (2025). Photocurrent generation
using ZnO/CuO/Ag heterojunction films. J. Mater.
Sci. Mater. Eng..

[36] Alhokbany N., Ahamad T., Alshehri S. M. (2022). Fabrication
of highly porous ZnO/Ag_2_O nanoparticles embedded in N-doped
graphitic carbon for photocatalytic
degradation of tetracycline. J. Environ. Chem.
Eng..

[37] Meena P. L., Poswal K., Surela A. K., Saini J. (2021). Facile synthesis of
ZnO/CuO/Ag_2_O ternary metal oxide nanocomposite for effective
photodegradation of organic water pollutants. Water Sci. Technol..

[38] Gade R., Basude M., Simhachalam N. B., V R.
D., Pola S., Chetti P. (2022). Synthesis of titanates for photomineralization of industrial
wastewater and organic pollutants. Environ.
Sci. Water Res. Technol..

[39] Alsulmi A., Mohammed N. N., Soltan A., Messih M. F. A., Ahmed M. A. (2023). Engineering
S-scheme CuO/ZnO heterojunctions sonochemically for eradicating RhB
dye from wastewater under solar radiation. RSC
Adv..

[40] Huang M., Yu J., Li B., Deng C., Wang L., Wu W., Dong L., Zhang F., Fan M. (2015). Intergrowth and coexistence
effects of TiO_2_-SnO_2_ nanocomposite with excellent
photocatalytic activity. J. Alloys Compd..

[41] Hassan S. M., Ahmed A. I., Mannaa M. A. (2019). Surface
acidity, catalytic and photocatalytic
activities of new type H_3_PW_12_O_40_/Sn-TiO_2_ nanoparticles. Colloids Surfaces A
Physicochem. Eng. Asp..

[42] Hezma A. M., Rajeh A., Mannaa M. A. (2019). An insight
into the effect of zinc
oxide nanoparticles on the structural, thermal, mechanical properties
and antimicrobial activity of Cs/PVA composite. Colloids Surfaces A Physicochem. Eng. Asp..

[43] Chang X., Li Z., Zhai X., Sun S., Gu D., Dong L., Yin Y., Zhu Y. (2016). Efficient synthesis of sunlight-driven ZnO-based heterogeneous
photocatalysts. Mater. Des..

[44] Zhai H., Wang L., Sun D., Han D., Qi B., Li X., Chang L., Yang J. (2015). Direct sunlight responsive
Ag-ZnO
heterostructure photocatalyst: Enhanced degradation of rhodamine B. J. Phys. Chem. Solids.

[45] Lemine O. M., Modwi A., Houas A., Dai J. H., Song Y., Alshammari M., Alanzi A., Alhathlool R., Bououdina M. (2018). Room temperature
ferromagnetism in Ni, Fe and Ag co-doped
Cu–ZnO nanoparticles: an experimental and first-principles
DFT study. J. Mater. Sci. Mater. Electron..

[46] Gupta R., Krishnarao Eswar N., Modak J. M., Madras G. (2016). Visible light driven
efficient N and Cu co-doped ZnO for photoinactivation of: Escherichia
coli. RSC Adv..

[47] Naseer S., Aamir M., Mirza M. A., Jabeen U., Tahir R., Malghani M. N. K., Wali Q. (2022). Synthesis of Ni-Ag-ZnO solid solution
nanoparticles for photoreduction and antimicrobial applications. RSC Adv..

[48] Alam U., Khan A., Raza W., Khan A., Bahnemann D., Muneer M. (2017). Highly efficient Y
and V co-doped ZnO photocatalyst
with enhanced dye sensitized visible light photocatalytic activity. Catal. Today.

[49] Jongnavakit P., Amornpitoksuk P., Suwanboon S., Ndiege N. (2012). Preparation and photocatalytic
activity of Cu-doped ZnO thin films prepared by the sol-gel method. Appl. Surf. Sci..

[50] Zhu H. D., Wang S., Wang S., Feng X. Q., Wang H., Zhang Q. J., Zhao Y. H. (2025). Photothermal catalytic hydrogen production
via methanol steam reforming on Cu/ZnO composites. Int. J. Hydrogen Energy.

[51] Barman D., Borah J., Deb S., Sarma B. K. (2023). Design strategy
for CuO-ZnO S-scheme heterojunction photocatalysts in the presence
of plasmonic Ag and insights into photoexcited carrier generation
and interfacial transfer in diverse structural configurations of the
heterostructure system. Colloids Surfaces A
Physicochem. Eng. Asp..

[52] Shirzad-Siboni M., Jonidi-Jafari A., Farzadkia M., Esrafili A., Gholami M. (2017). Enhancement
of photocatalytic activity of Cu-doped ZnO nanorods for the degradation
of an insecticide: Kinetics and reaction pathways. J. Environ. Manage..

[53] Chen S., Zhang L., Alshammari D. A., Hessien M. M., Yu W., Cui L., Ren J., El-Bahy Z. M., Guo Z. (2025). Z-scheme Ag2O/ZnO heterostructure
on carbon fibers for efficient photocatalysis of tetracycline. Sep. Purif. Technol..

[54] Wang X., Chen Q., Yang D., Li Y., Yin D., Ni B., Lu W., Sun G., Feng M. (2025). Ag doped ZnO hollow
nanosphere as a photocatalyst for enhanced performance in photo-assisted
Li-O2 batteries. Surfaces and Interfaces.

[55] Kwon D., Kim J. (2020). Silver-doped ZnO for photocatalytic
degradation of methylene blue. Korean J. Chem.
Eng..

[56] Alghamdi H. M., Abutalib M. M., Mannaa M. A., Nur O., Abdelrazek E. M., Rajeh A. (2022). Modification and development
of high bioactivities and environmentally
safe polymer nanocomposites doped by Ni/ZnO nanohybrid for food packaging
applications. J. Mater. Res. Technol..

[57] Hassan S. M., Ahmed A. I., Mannaa M. A. (2018). Structural,
photocatalytic, biological
and catalytic properties of SnO2/TiO2 nanoparticles. Ceram. Int..

[58] Huong L. M., Nam N. T. H., Dat N. T., Dat N. M., Cong C. Q., Ngan L. T., Ngan H. T. K., An H., Tai L. T., Hung P. N. P., Duy H. K., Minh N. C. A., Hai N. D., Tinh N. T., Thy L. T. M., Hieu N. H. (2023). Antibacterial mechanism
of phyto-synthesized CuO-decorated ZnO nanostructure in relation to
hydrogen peroxide generation under visible-light condition. Surfaces and Interfaces.

[59] Rahimpour R., Chaibakhsh N., Zanjanchi M. A., Moradi-Shoeili Z. (2020). Fabrication
of ZnO/FeVO_4_ heterojunction nanocomposite with high catalytic
activity in photo-Fenton-like process. J. Alloys
Compd..

[60] Buvaneswari K., Karthiga R., Kavitha B., Rajarajan M., Suganthi A. (2015). Effect of FeWO 4 doping on the photocatalytic
activity
of ZnO under visible light irradiation. Appl.
Surf. Sci..

[61] Bouzid H., Faisal M., Harraz F. A., Al-Sayari S. A., Ismail A. A. (2015). Synthesis of mesoporous Ag/ZnO nanocrystals
with enhanced
photocatalytic activity. Catal. Today.

[62] Dinesh V. P., Biji P., Kumaravel M., Tyagi A. K., Kamaruddin M. (2012). Synthesis
and characterization of hybrid zno@ag core-shell nanospheres for gas
sensor applications. Mater. Sci. Forum.

[63] Vivek C., Balraj B., Thangavel S. (2019). Structural,
optical and electrical
behavior of ZnO@Ag core–shell nanocomposite synthesized via
novel plasmon-green mediated approach. J. Mater.
Sci. Mater. Electron..

[64] Bruno E., Haris M., Mohan A., Senthilkumar M. (2021). Temperature
effect on CuO nanoparticles via facile hydrothermal approach to effective
utilization of UV–visible region for photocatalytic activity. Appl. Phys. A Mater. Sci. Process..

[65] Meshram S. P., Adhyapak P. V., Amalnerkar D. P., Mulla I. S. (2016). Cu doped ZnO microballs
as effective sunlight driven photocatalyst. Ceram. Int..

[66] Rahimi, R. ; Shokrayian, J. ; Rabbani, M. ;, Photocatalytic removing of methylene blue by using of Cu-doped ZnO, Ag-doped ZnO and Cu,Ag-codoped ZnO nanostructures, (2013) b019. DOI: 10.3390/ecsoc-17-b019.

[67] Raza W., Faisal S. M., Owais M., Bahnemann D., Muneer M. (2016). Facile fabrication of highly efficient
modified ZnO
photocatalyst with enhanced photocatalytic, antibacterial and anticancer
activity. RSC Adv..

[68] Uhm Y. R., Sun Han B., Rhee C. K., Choi S. J. (2013). Photocatalytic characterization
of Fe-and Cu-doped ZnO nanorods synthesized by cohydrolysis. J. Nanomater..

[69] Bousslama W., Elhouichet H., Férid M. (2017). Enhanced photocatalytic activity
of Fe doped ZnO nanocrystals under sunlight irradiation. Optik.

[70] Kaneva N. V., Dimitrov D. T., Dushkin C. D. (2011). Effect
of nickel doping on the photocatalytic
activity of ZnO thin films under UV and visible light. Appl. Surf. Sci..

[71] Phakathi N. A., Tichapondwa S. M., Chirwa E. M. N. (2025). Enhanced photodegradation of tetracycline
by novel porous g-C3N4 nanosheets under visible light irradiation. J. Photochem. Photobiol. A Chem..

[72] Phakathi N. A., Tichapondwa S. M., Chirwa E. M. N. (2022). Photocatalytic Degradation of Tetracycline
using Visible-light-driven Porous g- C3N4 Nanosheets Catalyst. Chem. Eng. Trans..

