# Supplementary material for: Impact of Heterovalent Cu2+ and Ag+ Doping on the Structural, Optoelectronic, and Photocatalytic Properties of ZnO for Enhanced Solar-Driven Hydrogen Evolution and Organic Pollutant Degradation
Source: ACS Omega. 2025 Sep 15;10(37):42847–61. doi: 10.1021/acsomega.5c05419 (PMC12461330; doi:10.1021/acsomega.5c05419)
Supplement: Supplementary file 1 [file ao5c05419_si_001.pdf]

Impact of Heterovalent Cu^{2+} and Ag^{+} Doping on the Structural, Optoelectronic, and Photocatalytic Properties of ZnO for Enhanced Solar-Driven Hydrogen Evolution and Organic Pollutant Degradation

Saedah R. Al-Mhyawi [1], Ahlam I. Al-Sulami [1*], Fatimah Mohammad H. AlSulami [1], Reema H. Aldahiri [1], Merfat M. Alsabban[1], Fuad Mohammed A. B. Mosa[2], Jawza Sh. Alnawmasi [3], Omer Nur[4], A. Rajeh [5], Mohammed A. Mannaa [6*],

[1] Department of Chemistry, College of Science, University of Jeddah, Jeddah, Saudi Arabia.

[2]Ministry of Energy, General Director for International Project Management Office and Advisor of Energy Minister, Saudi Arabia.

[3]Department of Chemistry, College of Science, Qassim University, Buraydah, 51452, Qassim, Saudi Arabia.

[4]Department of Science and Technology, Linkoping University, Campus Norrkoping, SE-60174, Norrkoping, Sweden.

[5]Physics Department, Faculty of Applied Science, Amran University, Sana'a, Yemen

[6]Chemistry Department, Faculty of Science, Amran University, Yemen

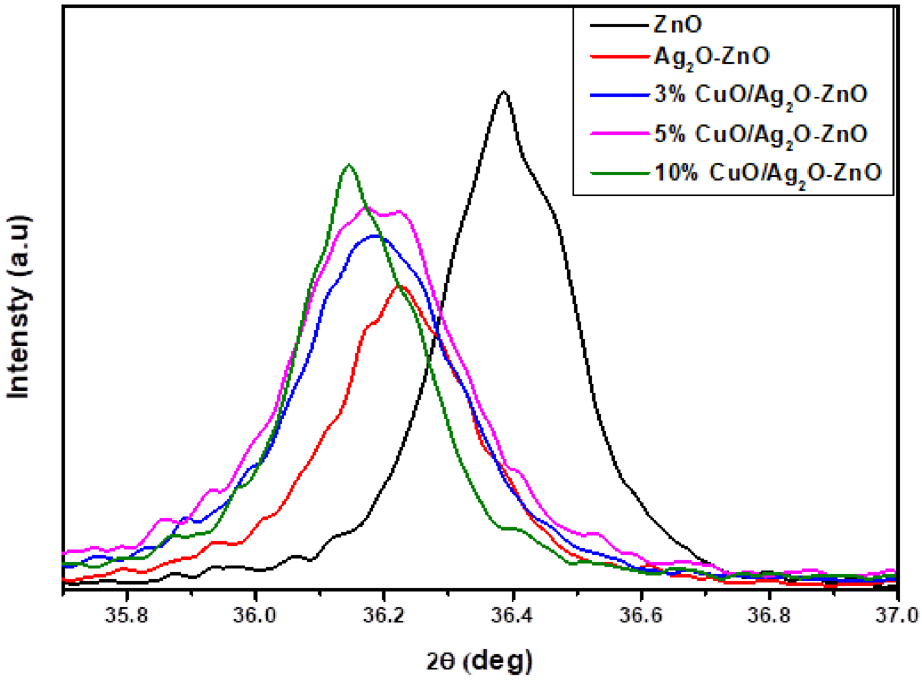

Figure S1: XRD patterns of pure and doped ZnO nanoparticles.

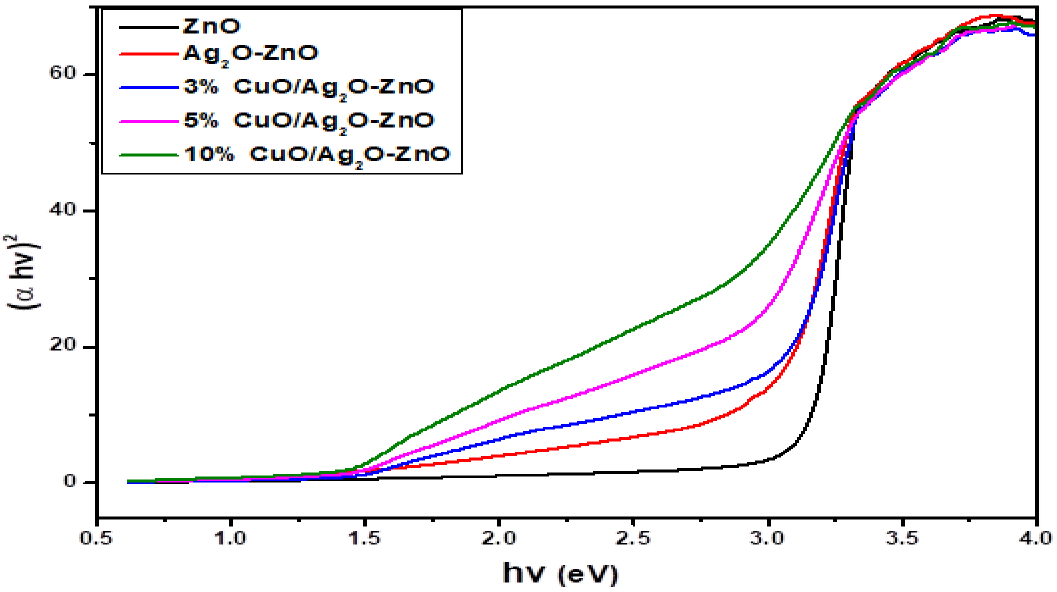

Figure S2: Band gap energy of pure and doped ZnO nanoparticles.

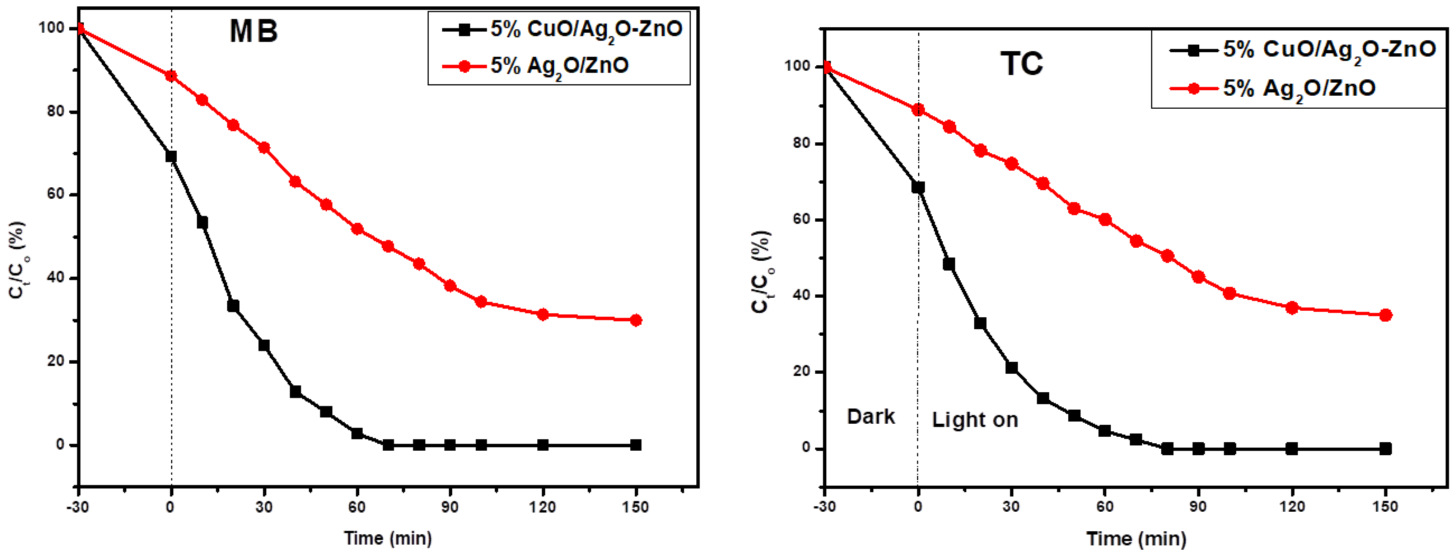

Figure S3. Photodegradation performance of MB and TC over 5%CuO/Ag_2O-ZnO and 5%Ag_2O-ZnO nanoparticles.

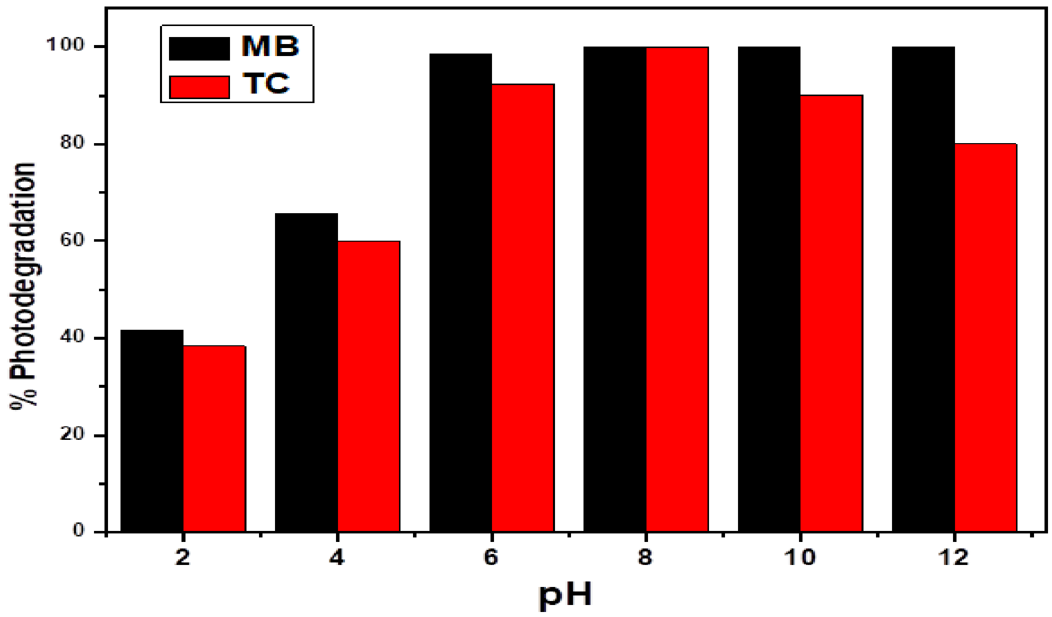

Figure S4. Effect of reaction solution pH on photodegradation of MB and TC over $5\%CuO/Ag_2O\text{-}ZnO$ heterostructure.

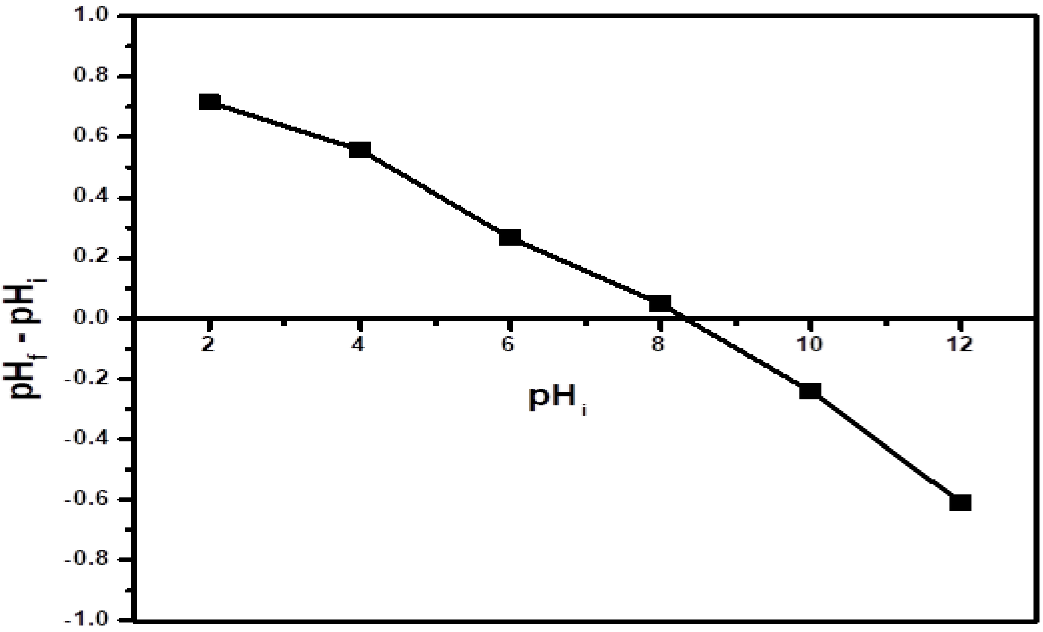

Figure S5. Determination of zero potential charge of $5\%CuO/Ag_2O\text{-}ZnO$ heterostructure.

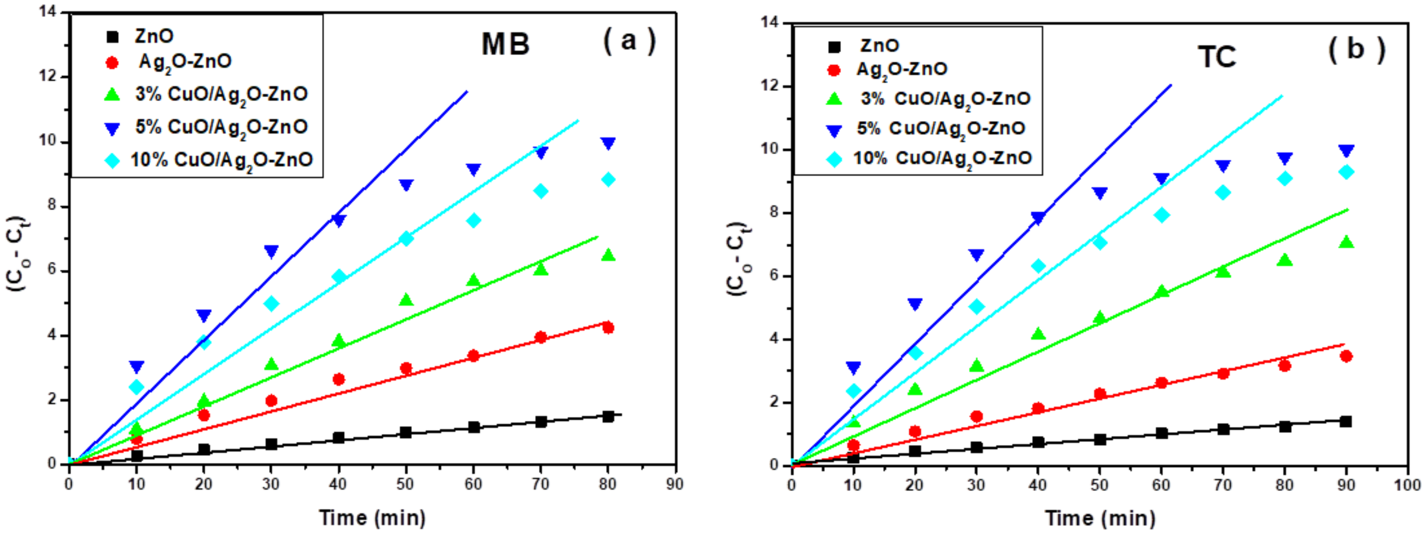

Figure S6. Linear plots of pseudo-zero-order kinetic model of (a) MB and (b) TC photodegradation on 5%CuO/Ag_2O-ZnO heterostructure.

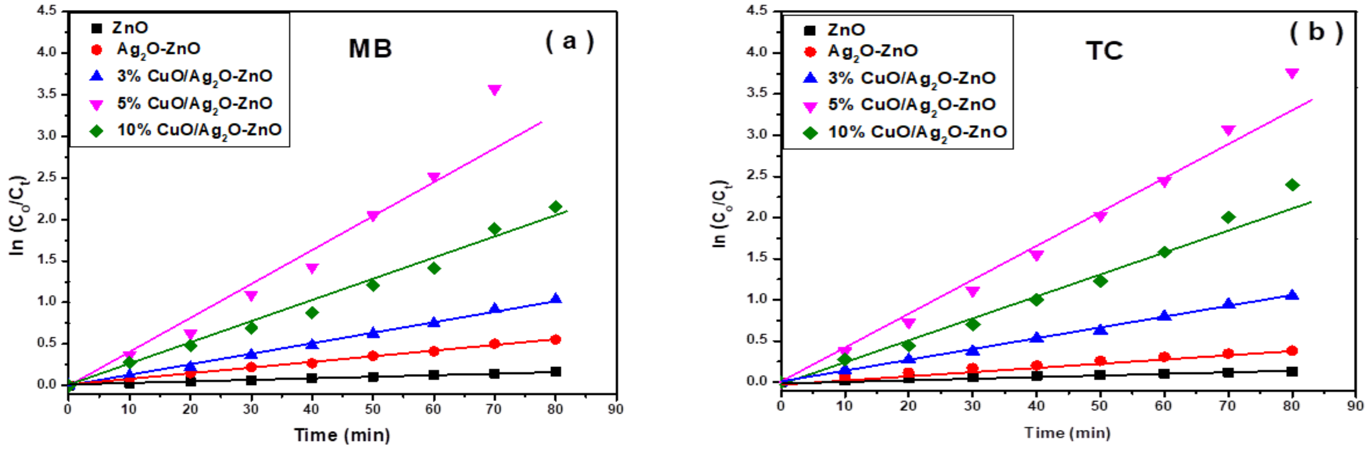

Figure S7. Linear Plots of pseudo-first-order kinetic model of (a) MB and (b) TC photodegradation on 5%CuO/Ag_2O-ZnO heterostructure.

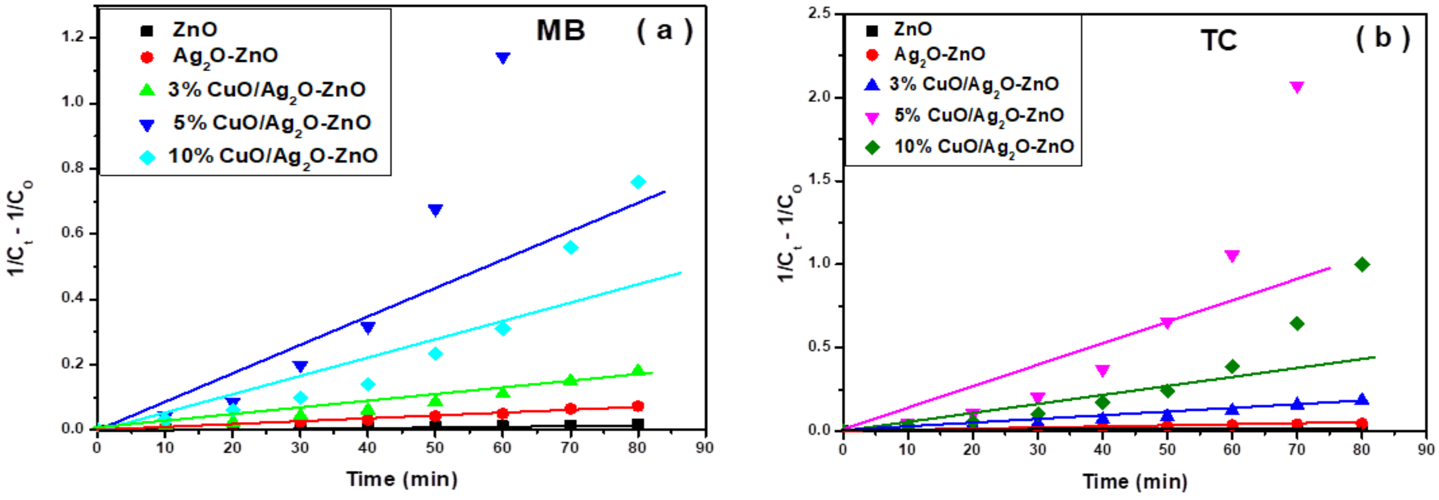

Figure S8. Linear plots of pseudo-second-order kinetic model of (a) MB and (b) TC photodegradation on 5%CuO/Ag_2O-ZnO heterostructure.

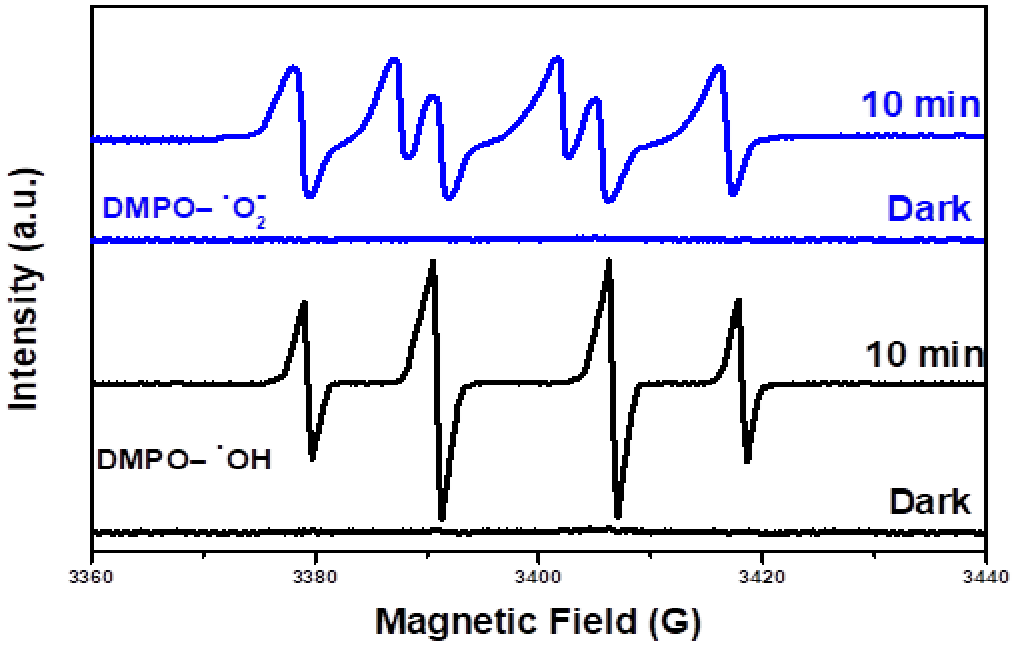

Figure S9. ESR spectra of 5% CuO/Ag_2O-ZnO heterostructure for •OH and •O_2^- radicals.

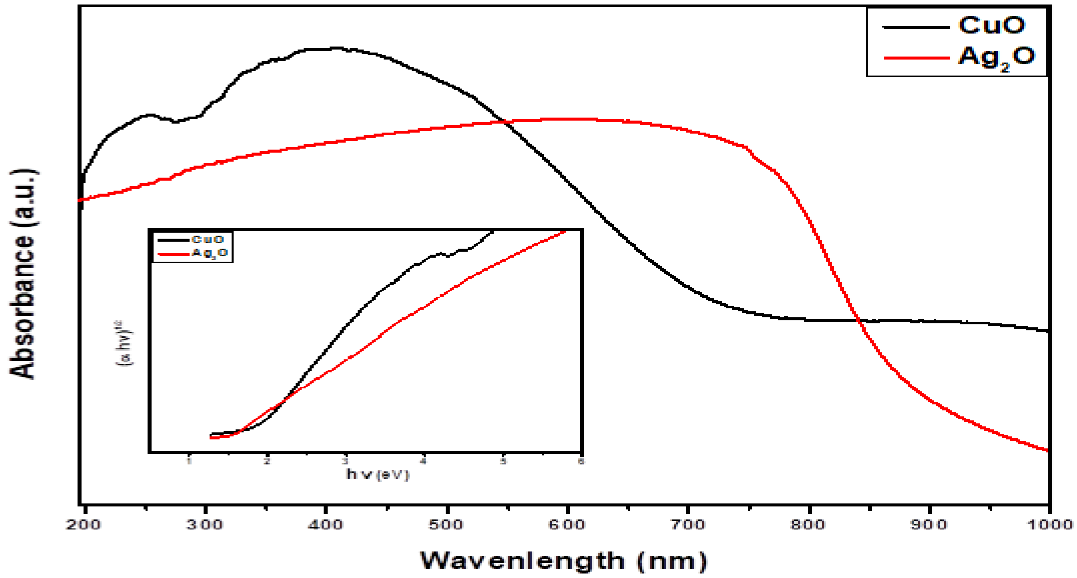

Figure S10. Uv-Vis spectra and band gap energy (inset) of CuO and Ag_2O nanoparticles.

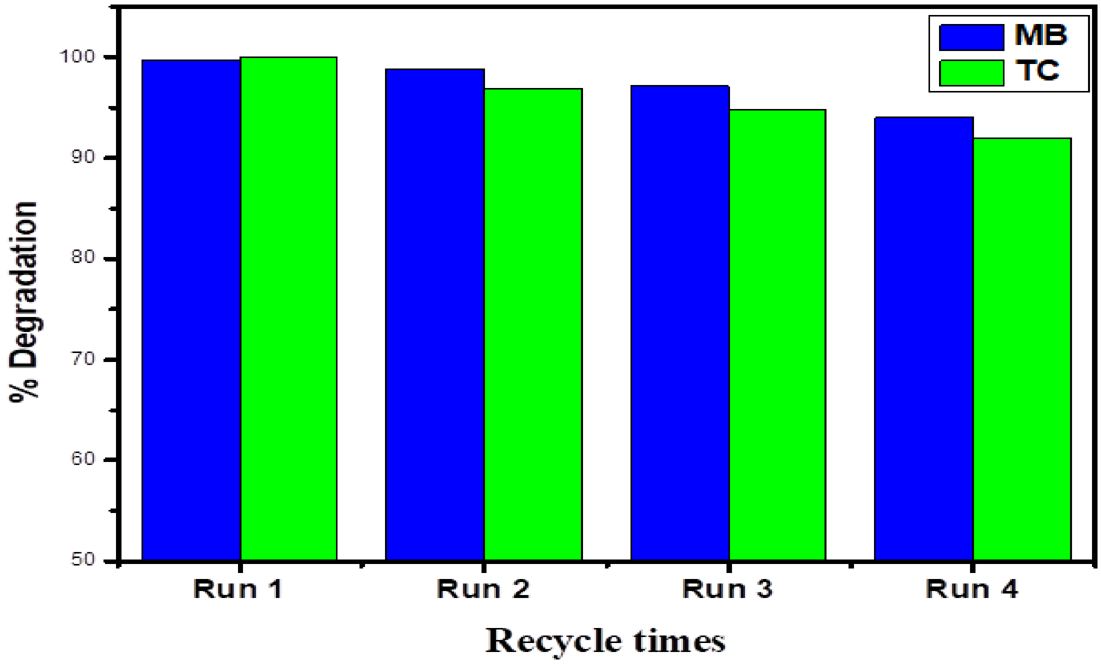

Figure S11. Effect of recycle times on the photocatalytic performance of 5% CuO/Ag_2O-ZnO heterostructure.

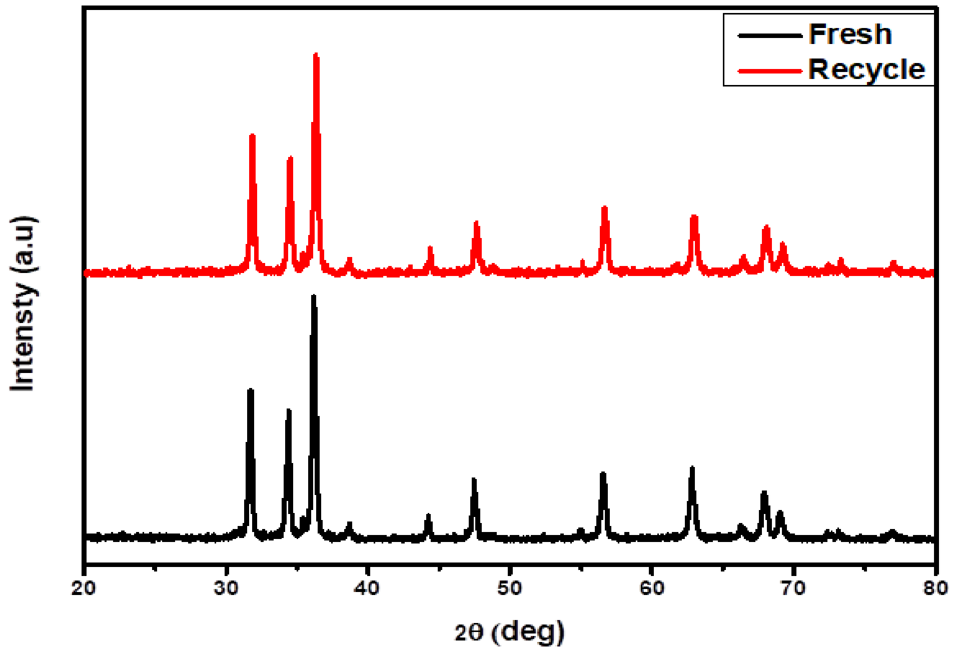

Figure S12. Effect of recycle on the structural properties of 5%CuO/Ag_2O-ZnO heterostructure.

Table S1. Kinetic parameters of zero-order model for MB and TC photodegradation

Samples	MB		TC	
	K_0 (mg^{-1}·L·min^{-1})	R^2	K_0 (mg^{-1}·L·min^{-1})	R^2
ZnO	0.0193	0.9977	0.0163	0.9918
Ag_2O-ZnO	0.0566	0.9948	0.0418	0.9909
3% CuO/Ag_2O-ZnO	0.0871	0.9943	0.0864	0.9877
5% CuO/Ag_2O-ZnO	0.1517	0.9474	0.1397	0.9389
10% CuO/Ag_2O-ZnO	0.1268	0.9659	0.1228	0.9715

Table S2. Kinetic parameters of second-order model for MB and TC photodegradation.

Samples	MB		TC	
	K_2 (L·mg^{-1}· min^{-1})	R^2	K_2 (L·mg^{-1}· min^{-1})	R^2
ZnO	0.0002	0.9925	0.0009	0.9950
Ag_2O-ZnO	0.0009	0.9859	0.0006	0.9899
3% CuO/Ag_2O-ZnO	0.0019	0.9767	0.0021	0.9732
5% CuO/Ag_2O-ZnO	0.0261	0.6425	0.0296	0.7128
10% CuO/Ag_2O-ZnO	0.0068	0.8855	0.0084	0.8511

Table S3. Comparison of the photodegradation efficiency of our photocatalysts with different photocatalysts reported in the literatures.

Catalyst	Type of pollutant	Light source	Time, (min.)	Efficiency (%)	Ref.
**CuO/Ag_2O-ZnO**	MB	Sunlight	60	100	This work
	TC	Sunlight	80	100	
**ZnO/CuO**	RhB	Visible	120	93	[1]
**ZnO/CuO/Ag**	MO	visible	110	75	[2]
**CuO-ZnO**	MB	UV	80	98.6	[3]
**Fe_3O_4/Co_3O_4-TiO_2**	TC	visible	120	100%	[4]
**ZnO/Ag2O**	TC	visible	60	90.7	[5]
**RGO/Fe_3O_4–$FeVO_4$**	MB	Sunlight	180	100	[6]
**Ag-ZnO**	MB	Sunlight	180	92.7	[7]
**CuO/ZnO**	MB	Sunlight	150	95	[8]

References

[1] D.T. Nguyen, M.D. Tran, T. Van Hoang, D.T. Trinh, D.T. Pham, D.L. Nguyen, Experimental and numerical study on photocatalytic activity of the ZnO nanorods/CuO composite film, Sci. Rep. 10 (2020) 1–9. https://doi.org/10.1038/s41598-020-64784-w.

[2] R. Patwa, S. Rohilla, Jyoti saini, N. Goel, Structural and spectroscopy analysis of nanocomposites of metal oxide ZnO/CuO/Ag by coprecipitation: Potential

application in photocatalysis, Ceram. Int. (2025). https://doi.org/10.1016/j.ceramint.2025.01.242.

[3] K.P. Sapkota, I. Lee, S. Shrestha, A. Islam, A. Hanif, J. Akter, J.R. Hahn, Coherent CuO-ZnO nanobullets maneuvered for photocatalytic hydrogen generation and degradation of a persistent water pollutant under visible-light illumination, J. Environ. Chem. Eng. 9 (2021) 106497. https://doi.org/10.1016/j.jece.2021.106497.

[4] M.M. Abutalib, H.M. Alghamdi, A. Rajeh, O. Nur, A.M. Hezma, M.A. Mannaa, Fe3O4/Co3O4-TiO2S-scheme photocatalyst for degradation of organic pollutants and H2production under natural sunlight, J. Mater. Res. Technol. 20 (2022) 1043–1056. https://doi.org/10.1016/j.jmrt.2022.07.078.

[5] N. Alhokbany, T. Ahamad, S.M. Alshehri, Fabrication of highly porous ZnO/Ag2O nanoparticles embedded in N-doped graphitic carbon for photocatalytic degradation of tetracycline, J. Environ. Chem. Eng. 10 (2022) 107681. https://doi.org/10.1016/j.jece.2022.107681.

[6] Q.A. Alsulami, A. Rajeh, M.A. Mannaa, S.M. Albukhari, D.F. Baamer, One-step preparation of RGO/Fe3O4–FeVO4 nanocomposites as highly effective photocatalysts under natural sunlight illumination, Sci. Rep. 12 (2022) 1–12. https://doi.org/10.1038/s41598-022-10542-z.

[7] D. Kwon, J. Kim, Silver-doped ZnO for photocatalytic degradation of methylene blue, Korean J. Chem. Eng. 37 (2020) 1226–1232. https://doi.org/10.1007/s11814-020-0520-7.

[8] T. Tangcharoen, W. Klysubun, C. Kongmark, Synthesis and characterization of nanocrystalline CuO/ZnO composite powders with enhanced photodegradation performance under sunlight irradiation, J. Mater. Sci. Mater. Electron. 31 (2020) 12807–12822. https://doi.org/10.1007/s10854-020-03834-5.
